# Disparate roles for *C. elegans* DNA translocase paralogs RAD-54.L and RAD-54.B in meiotic prophase germ cells

**DOI:** 10.1093/nar/gkad638

**Published:** 2023-08-07

**Authors:** Kei Yamaya, Bin Wang, Nadin Memar, Arome Solomon Odiba, Alexander Woglar, Anton Gartner, Anne M Villeneuve

**Affiliations:** Department of Developmental Biology, Stanford University School of Medicine, Stanford, CA, USA; State Key Laboratory of Non-food Biomass and Enzyme Technology, Guangxi Academy of Sciences, 530007 Nanning, China; IBS Center for Genomic Integrity and Department for Biological Sciences, Ulsan National Institute of Science and Technology, Ulsan, Korea; State Key Laboratory of Non-food Biomass and Enzyme Technology, Guangxi Academy of Sciences, 530007 Nanning, China; Department of Developmental Biology, Stanford University School of Medicine, Stanford, CA, USA; Swiss Institute for Experimental Cancer Research (ISREC) and School of Life Sciences, Swiss Federal Institute of Technology Lausanne (EPFL), Lausanne, Switzerland; IBS Center for Genomic Integrity and Department for Biological Sciences, Ulsan National Institute of Science and Technology, Ulsan, Korea; Department of Developmental Biology, Stanford University School of Medicine, Stanford, CA, USA; Department of Genetics, Stanford University School of Medicine, Stanford, CA, USA

## Abstract

RAD54 family DNA translocases partner with RAD51 recombinases to ensure stable genome inheritance, exhibiting biochemical activities both in promoting recombinase removal and in stabilizing recombinase association with DNA. Understanding how such disparate activities of RAD54 paralogs align with their biological roles is an ongoing challenge. Here we investigate the *in vivo* functions of *Caenorhabditis elegans* RAD54 paralogs RAD-54.L and RAD-54.B during meiotic prophase, revealing distinct contributions to the dynamics of RAD-51 association with DNA and to the progression of meiotic double-strand break repair (DSBR). While RAD-54.L is essential for RAD-51 removal from meiotic DSBR sites to enable recombination progression, RAD-54.B is largely dispensable for meiotic DSBR. However, RAD-54.B is required to prevent hyperaccumulation of RAD-51 on unbroken DNA during the meiotic sub-stage when DSBs and early recombination intermediates form. Moreover, DSB-independent hyperaccumulation of RAD-51 foci in the absence of RAD-54.B is RAD-54.L-dependent, revealing a hidden activity of RAD-54.L in promoting promiscuous RAD-51 association that is antagonized by RAD-54.B. We propose a model wherein a division of labor among RAD-54 paralogs allows germ cells to ramp up their capacity for efficient homologous recombination that is crucial to successful meiosis while counteracting potentially deleterious effects of unproductive RAD-51 association with unbroken DNA.

## Graphical Abstract

**Graphical Abstract:**
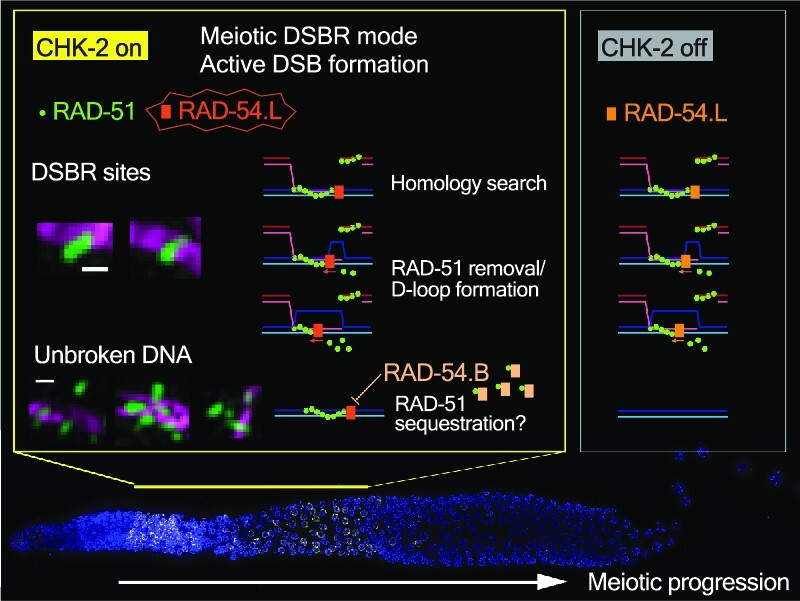
*Caenorhabditis elegans* RAD-54.L and RAD-54.B have distinct roles during the meiotic prophase substage when protein kinase CHK-2 is active. We propose that hyperactivation of RAD-54.L during this stage enables efficient repair of the many programmed meiotic DSBs that are formed, but also boosts an ability of RAD-54.L to promote/stabilize unproductive association of RAD-51 with unbroken DNA. RAD-54.B antagonizes this off-target binding, possibly by sequestering RAD-51 in the nucleoplasm.

## INTRODUCTION

Homologous recombination plays a central role in ensuring the faithful inheritance of genomes during meiosis, the specialized cell division program by which diploid organisms generate haploid gametes. In most sexually reproducing organisms, the formation of at least one crossover (CO) between each chromosome pair is crucial for faithful segregation of homologous chromosomes at the first meiotic division. To produce this obligate CO, many programmed double-strand DNA breaks (DSBs) are introduced into the genome by the meiosis-specific SPO11 protein complex and are subsequently repaired through homologous recombination (1). In most organisms studied, only a subset of DSB repair (DSBR) sites mature into COs; COs are often limited to one or a few per chromosome pair (or per chromosome arm), with the majority of introduced DSBs being repaired as non-COs (NCOs) (2). Thus, both DSB formation and repair occur within a highly regulated framework that must simultaneously ensure both CO formation and restoration of genome integrity.

Recombinases play crucial roles in the early steps of homologous recombination. Following induction and processing of DSBs to yield 3′ single-stranded overhanging ends, recombinases are recruited to DSBR sites (3–5) and form nucleoprotein filaments on the resected 3′ ssDNA, where they promote the search for a homologous DNA repair template and subsequently facilitate strand invasion into the template DNA duplex (6,7). Most eukaryotes have two recombinases, RAD51, which is widely expressed in both somatic and germ cells, and DMC1, which is specialized to function in meiotic recombination, reflecting an ancient duplication of the ancestral recombinase gene early in the eukaryotic lineage (8–10). However, DMC1 has been lost in several eukaryotic lineages, including many Hymenopteran and Dipteran insects and the nematode sublineage that includes the Caenorhabditis genus and its close relatives (10,11). Thus, RAD-51 is the sole recombinase acting in both mitotically-cycling cells and meiotic prophase nuclei in the *Caenorhabditis elegans* germ line.

Eukaryotic recombinases RAD51 and DMC1 function in partnership with RAD54 family DNA translocases, which are members of the Snf2/Swi2 group of ATP-dependent DNA motors (6,12). RAD54 family proteins have a conserved C-terminal ATPase/helicase-like domain that promotes translocation along double-stranded (ds) DNA and a more variable N-terminal domain that mediates interactions with recombinase partners and is required for multiple aspects of RAD54 function (6,12).

RAD54L orthologs, represented by Rad54 in *Saccharomyces cerevisiae* and previously called RAD54 or RAD54A in the mammalian literature, have been studied extensively through both biochemical and cell-based assays (6,12). *In vitro*, RAD54(L) has been demonstrated to have dsDNA-stimulated ATPase activity (13), ATP-dependent dsDNA translocase activity (14), and ATP-dependent chromatin remodeling activity (15–17). Further, numerous *in vitro* assays have demonstrated several activities relevant to RAD51 recombinase-mediated homologous recombination. These include stabilization of RAD51 association with ssDNA (18), removal of RAD51 filaments from dsDNA (mediated by RAD54 translocation) (19), and stimulation of RAD51-mediated formation of D-loops (key DSBR intermediates resulting from successful strand invasion) (20), with this latter activity likely reflecting contributions of both RAD51 stabilization and removal activities (6,12). In addition, *invitro* single-molecule imaging assays suggest that the RAD51-filament stabilizing and dsDNA translocase activities of RAD54(L) may act together to enhance the efficiency of RAD51-mediated homology search by enabling motor-driven one-dimensional translocation of RAD51-ssDNA nucleoprotein filaments along dsDNA (21,22). *In vivo* studies support the biological importance of a close functional partnership between RAD54L orthologs and RAD51 in maintaining genome integrity. *S. cerevisiae* Rad54 is required for DNA repair after damage in mitotic cells, with mutants exhibiting a phenotype identical to *rad51* mutants (23,24), and mammalian RAD54 is required for homologous recombination in mammalian cells (25). Further, cytological evidence from mammalian cells supports RAD54 having both an ATP-independent activity that promotes efficient recruitment of RAD51 to DSBR sites and an ATP-dependent activity that promotes RAD51 removal (26,27). In *C. elegans* meiocytes, RAD-54.L (formerly known as RAD-54) has been shown to be essential for removal of RAD-51 from DSBR sites (28–31).

Many eukaryotes have a second RAD54 paralog, exemplified by Rdh54/Tid1 in *S. cerevisiae* and RAD54B in mammals (12). The phylogenetic distribution of RAD54 paralogs is consistent with the possibility that separate RAD54L/Rad54 and RAD54B/Rdh54 paralogs originated from an ancient gene duplication that predated the divergence of plants, animals, and fungi (11, 12, WormBase website, release WS286 (2022), http://www.wormbase.org). In contrast to RAD54L, where clear orthologs are present throughout the eukaryotic lineage, apparent RAD54B/Rdh54 orthologs are absent from some lineages and exhibit greater sequence diversification than RAD54L in lineages where they are present. We speculate that duplication of an ancestral RAD54 gene to yield separate RAD54L and RAD54B paralogs may have been coupled to duplication of the ancestral recombinase RAD51 gene to yield the meiosis-specific recombinase DMC1. However, this is neither an essential nor exclusive partnership, as RAD54B is absent from many plant lineages and some arthropod lineages that retain DMC1 (11), and RAD54B is present in Caenorhabditis nematodes despite the loss of DMC1 (WormBase website, release WS286 (2022), http://www.wormbase.org).

Genetic and biochemical studies have provided evidence for both distinct and partially overlapping roles for RAD54L and RAD54B homologs. Biochemically, Rad54 and Rdh54 have been shown to be very similar, with some differences in their ATPase activity and translocation velocity and processivity (6,12,32,33). However, genetic evidence indicates a significant division of labor between the two paralogs: *e.g*. the *S. cerevisiae rad54* mutant primarily exhibits defects in mitotic DSBR, while the *rdh54* mutant primarily exhibits defects in meiotic DSBR (34–36). Consistent with these divergent mutant phenotypes, biochemical evidence supports Dmc1 preferentially functioning together with Rdh54, and Rad51 preferentially functioning with Rad54 (37,38). Despite this clear evidence for specialization, however, multiple studies also indicate partial functional redundancy between the two paralogs. For example, the yeast *rad54 rdh54* double mutant exhibits stronger defects in both mitosis and meiosis compared to either single mutant (34). Moreover, additional functions for *S. cerevisiae* Rdh54 and mammalian RAD54B have also been demonstrated in mitotically dividing cells where DMC1 is absent and RAD51 is the only recombinase. For example, yeast Rdh54 has been implicated in limiting the size of Rad51-mediated D-loop intermediates *in vivo* (39,40), and biochemical evidence indicates that the two RAD54 paralogs may occupy different sites on assembled presynaptic filaments (41), suggesting that both RAD54 paralogs may be deployed in different ways at the same DSBR site. Further, loss of either RAD54 paralog causes increased sensitivity to DNA damaging agents both in mouse embryonic stem cells and mice, with loss of both paralogs causing stronger sensitivity (42). Additionally, RAD54B orthologs have been shown to antagonize potentially toxic association of RAD51 with unbroken DNA in both mammalian cells and vegetatively growing yeast cells (43,44), paralleling a role for Rdh54 in antagonizing association of Dmc1 with unbroken DNA during yeast meiosis (45). Thus, while multiple disparate biochemical activities have been identified for RAD54 family proteins, much remains to be learned regarding which activities are employed *in vivo* and how each paralog may contribute differentially to DSBR in different biological contexts.

Here, we investigate the *in vivo* biological roles of RAD54 family paralogs RAD-54.L and RAD-54.B during meiosis in the nematode *C. elegans*. We demonstrate that RAD-54.L and RAD-54.B make distinct contributions to meiotic homologous recombination and provide evidence for disparate activities in regulating RAD-51 recombinase. While RAD-54.L is essential for the progression of meiotic DSBR, RAD-54.B is largely dispensable for the completion of meiotic recombination and instead functions in inhibiting the promiscuous accumulation of RAD-51 on unbroken DNA during the substage of meiotic prophase when meiotic DSBs and early meiotic recombination intermediates form. Unexpectedly, we found that hyperaccumulation of RAD-51 at unbroken DNA in the absence of RAD-54.B is dependent on RAD-54.L, a surprising *in vivo* demonstration of its activity to stabilize/promote RAD-51-DNA binding, indicating that RAD-54.L can both promote and antagonize RAD-51 binding in the same cells. Moreover, we found that RAD-54.B inhibits unproductive RAD-51 binding to unbroken DNA through an ATPase-independent mechanism. Taken together, our data suggest a model in which RAD-54.L is hyperactivated during meiotic prophase to accommodate an increased burden on the DSBR machinery imposed by programmed DSB induction. We further suggest that RAD-54.B may counteract the potentially deleterious effects of RAD-54.L hyperactivation by sequestering RAD-51 protomers to inhibit ectopic formation of RAD-51 filaments on unbroken DNA.

## MATERIALS AND METHODS

### *C*.*elegans* strains

The following *C. elegans* strains were used and are available from AMV upon request.

AV695: *meIs8[pie-1p::GFP::cosa-1::unc-119(+)] II; mnT12 (X; IV)*
AV776: *spo-11(me44)/nT1 IV*
AV863: *nbs-1(me106)/mnC1 II*
AV1158: *him-6(jf93[him-6::HA]) rad-54.B(gt3402[rad-54.B::GFP]) IV*
AV1179: *rad-54.L(me98)/tmC18[dpy-5(tmIs1236)] I*
AV1189: *spo-11(me44) rad-54.B(gk340656)/tmC5[F36H1.3(tmIs1220)] rad-54.B(gk340656) IV*
AV1190: *rad-54.L(me98)/tmC18[dpy-5(tmIs1236)] I; spo-11(me44) rad-54.B(vc205209)/tmC5[F36H1.3(tmIs1220)] rad-54.B(gk340656) IV*
AV1191: *rad-54.L(me98) I; opIs257 [rad-54.Lp::rad-54.L::YFP::rad-54.L 3′UTR + unc-119(+)]*
AV1196: *rad-54.L(me98)/tmC18[dpy-5(tmIs1236)] I; rad-54.B(gk340656) IV*
AV1199: *spo-11(me44)/tmC5[F36H1.3(tmIs1220)] IV*
AV1203: *rad-54.L(me98)/tmC18[dpy-5(tmIs1236)] I; spo-11(me44)/ tmC5[F36H1.3(tmIs1220)] IV*
AV1214: *rad-54.L(me98) I; opIs257 [rad-54.Lp::rad-54.L::YFP::rad-54.L 3′UTR + unc-119(+)]; rad-54.B (gk340656) IV*
AV1215: *rad-54.B(gk340656) IV; egl-1(n1084) chk-2(gk212)/egl-1(n1084) yIs34 oxTi633 V*
AV1216: *rad-54.B(gk340656) IV; egl-1(n1084) yIs34 oxTi633 V*
AV1238: *rad-54.L(me139)/tmC18[dpy-5(tmIs1236)] I; ieSi21[sun-1::mRuby] IV*
AV1239: *rad-54.L(me139)/tmC18[dpy-5(tmIs1236)] I; ieSi11[EmGFP::syp-3] II; rad-54.B(gk340656) IV*
AV1284: *rad-54.L(me177[K238R])/tmC18[dpy-5(tmIs1236)] I*
AV1292: *rad-54.L(me177[K238R])/tmC18[dpy-5(tmIs1236)] I; spo-11(me44) rad-54.B(gk340656)/ tmC5[F36H1.3(tmIs1220)] rad-54.B(gk340656) IV*
AV1311: *spo-11(me44) rad-54.B(gt3379[K244A])/tmC5 rad-54.B(gt3379) IV*
CB4856: *C. elegans* wild isolate ‘Hawaiian’
CB5584: *mIs12[myo-2p::GFP + pes-10p::GFP + F22B7.9p::GFP] II*
TG3319: *rad-54.B(gk340656*)
TG4580: *rad-54.B(gt3328) IV*
TG4080: *rad-54.B(gk340656) IV; meIs8[pie-1p::GFP::cosa-1::unc-119(+)] II*
TG4252: *rad-54.B(gk340656) IV; meIs8[pie-1p::GFP::cosa-1::unc-119(+)] II; mnT12 (X; IV)*
TG4685: *rad-54.B(gt3379[K244A])*
XSW933: r*ad-54.B(gk340656) IV; ‘Hawaiian’ V*. (This strain was derived from inbreeding following an intercross between TG3319 and CB4856 to yield a strain carrying the Hawaiian-derived alleles of chromosome V single nucleotide polymorphism (SNP) markers.)

### Antibodies

The following primary antibodies were used: chicken anti-HTP-3 (1:1000, (46)), chicken anti-GFP (1:250, (Abcam)), mouse anti-GFP (1:500, (Roche)), rabbit anti-GFP (1:500, (47)), mouse anti-HA (1:1000, (Clone 16B12; Covance)), rabbit anti-MSH-5 (1:10 000, (SDIX)), rabbit anti-RAD-51 (1:500, (48)), rat anti-RAD-51 (1:200, (49)), guinea pig anti-SUN-1 pS24 (1:700, (50)), guinea pig anti-SUN-1 pS8(1:1000, (50)), guinea pig anti-SYP-1 (1:200, (51)). Secondary antibodies used were Alexa Fluor 405 (1:100), 488 (1:400), 555 (1:400), and 647 (1:200)-conjugated goat antibodies raised against the appropriate species (Life Technologies).

### *C*.*elegans* culturing, genetics, and gene editing

**Figure 1.**
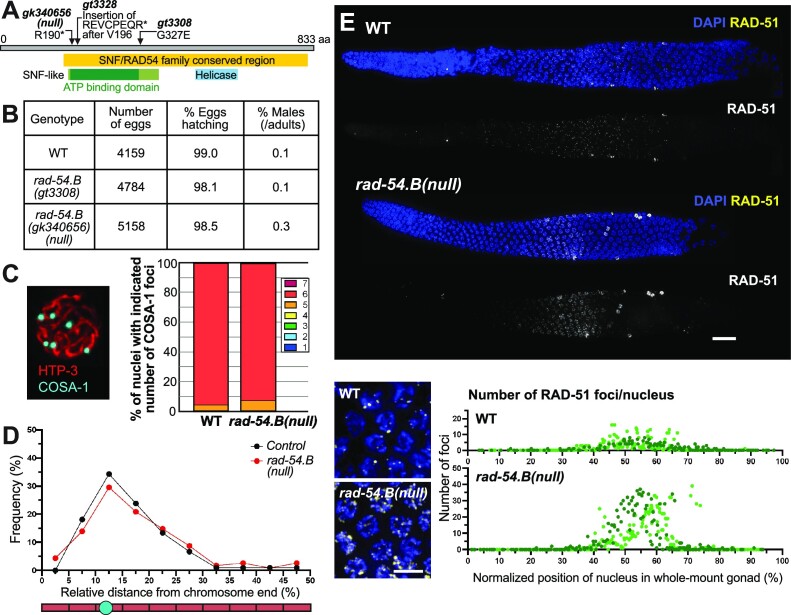
Loss of *rad-54.B* function is compatible with successful meiosis but causes temporary hyperaccumulation of RAD-51. (**A**) Diagram of RAD-54.B protein with alterations caused by mutant alleles; * indicates stop codon. Yellow box indicates region conserved across SNF/RAD54 family members. Green box indicates SNF2-like N-terminal domain, with dark green indicating the ATP-binding domain of helicase superfamily 1/2; blue box indicates C terminal helicase domain. Both *gk340656*, which introduces a stop codon at position 190 of the 833 amino acid coding sequence (R190*), and *gt3328*, a CRSPR-engineered mutation introducing a stop-in cassette, are expected to be null alleles, based on early termination of translation upstream of most of the SNF/RAD54 family conserved region coding sequence, accompanied by nonsense-mediated decay of the transcript. Unless otherwise noted, experiments were conducted using the *gk340656* presumed null allele. (**B**) Quantification of progeny viability and fraction of males among adult progeny, indicating successful meiotic chromosome segregation in *rad-54.B* mutants. Number of broods assayed: WT, *n* = 13; *rad-54.B(gt3308), n* = 16; *rad-54.B(gk340656), n* = 16. (**C**) Left: Example *meIs8[pie-1p::GFP::cosa-1::unc-119(+)]* nucleus immunostained for HTP-3 and GFP. Right: Quantification of COSA-1 foci in late pachytene nuclei from WT and *rad-54.B* gonads. Number of nuclei assayed: WT, *n* = 251; *rad-54.B, n* = 283. (**D**) Distribution of positions of CO site foci of control (*meIs8; mnT12*) and *rad-54.B* (*meIs8; rad-54.B mnT12*) late pachytene nuclei on non-fusion chromosomes, represented as relative distance from the nearest chromosome end. Control, *n* = 88 chromosomes; *rad-54.B, n* = 100 chromosomes. (**E**) Top: Max-projected images of WT and *rad-54.B* whole-mount gonads immunostained for RAD-51, showing elevated levels of RAD-51 foci detected in the *rad-54.B* mutant. Scale bar represents 20 μm. Bottom, left: zoomed-in fields of nuclei in zone of peak RAD-51 accumulation in both WT and *rad-54.B* gonads. Bottom, right: Quantification of RAD-51 foci in WT and *rad-54.B* gonads. Each data point represents an individual nucleus, with relative position calculated as the percentage of the length between the distal tip (0%) and the end of pachytene zone (100%); light and dark colors represent the two different gonads used for quantification.

**Figure 2.**
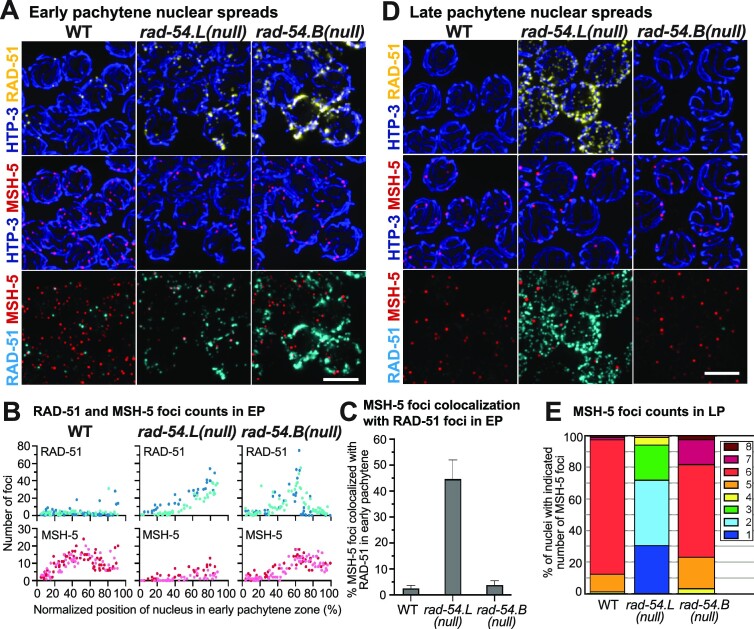
Differences in progression of meiotic recombination in *rad-54.L* and *rad-54.B* mutants. (**A**) Max-projected images of early pachytene nuclear spreads from WT, *rad-54.L(me98)*, and *rad-54.B(gk340656)* gonads, immunostained for RAD-51, meiotic recombination protein MSH-5 and chromosome axis protein HTP-3. Scale bar represents 5 μm. (**B**) Quantification of RAD-51 and MSH-5 foci numbers in early pachytene nuclei from spread gonads. Each data point represents an individual nucleus, showing its relative position within the early pachytene zone and number of RAD-51 or MSH-5 foci. Light and dark colors in the same plot represent the two different gonads used in quantification. In the *rad-54.B* mutant, MSH-5 foci accumulate with some delay, but foci numbers plateau at WT-like levels (WT versus *rad-54.B*, first half of early pachytene zone, *P* < 0.0001, second half of early pachytene zone, n.s). The *rad-54.L* mutant has reduced numbers of MSH-5 foci compared to WT and the *rad-54*.B mutant (WT vs *rad-54.L, P* < 0.0001 for either half of the early pachytene zone, *rad-54.B* vs *rad-54.L, P* < 0.0001 for either half of the early pachytene zone). Statistical significance was assessed using the Mann Whitney test. (**C**) Percentage of MSH-5 foci colocalizing with RAD-51 foci in early pachytene spread nuclei (total numbers of MSH-5 foci analyzed: WT, *n* = 1083; *rad-54.L, n* = 177; *rad-54.B, n* = 808). Error bars indicate 95% confidence interval. (**D**) Max-projected images of RAD-51, MSH-5 and HTP-3 immunostaining in late pachytene spread nuclei, illustrating approximately normal numbers of MSH-5 foci in the *rad-54.B* mutant and persistence of high levels of RAD-51 foci and reduced numbers of MSH-5 foci in the *rad-54.L* mutant. Scale bar represents 5 μm. (**E**) Quantification of MSH-5 foci in late pachytene nuclear spreads (numbers of nuclei analyzed: WT, *n* = 81; *rad-54.L, n* = 90; *rad-54.B, n* = 122). Analysis using Mann–Whitney test indicated that late pachytene MSH-5 foci counts in the *rad-54.B* mutant were overall comparable to WT, but were substantially reduced in the *rad-54.L* mutant (WT vs *rad-54.L, P* < 0.0001; *rad-54.B* versus *rad-54.L, P* < 0.0001; WT versus *rad-54.B*, n.s.) However, a categorical comparison (*i.e*. ‘< or > 6 foci’ versus exactly 6 foci) of WT versus *rad-54.B* by Fisher exact test did suggest an elevated occurrence of late pachytene nuclei with fewer or greater than six foci in the *rad-54.B* mutant (*P* < 0.0001).

**Figure 3.**
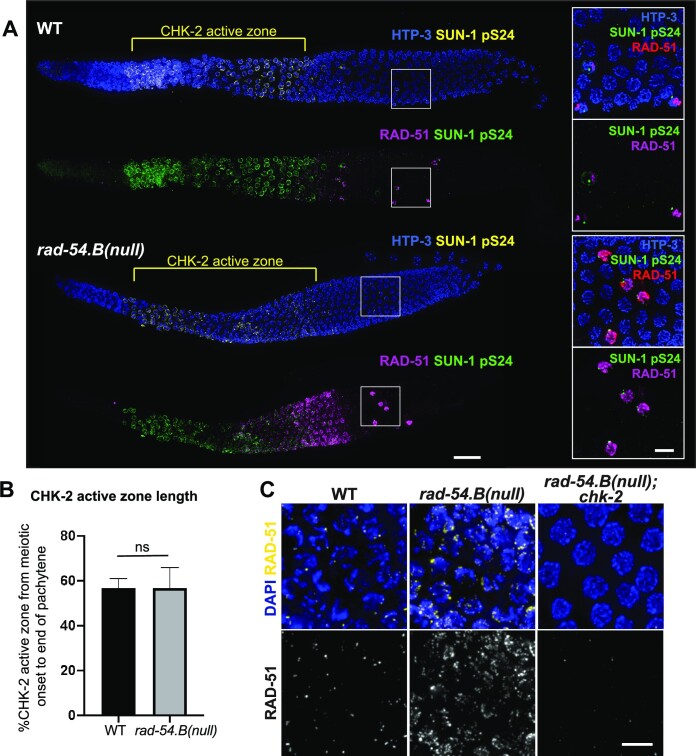
Relationship between CHK-2 activity and RAD-51 hyperaccumulation in the *rad-54.B* mutant. (**A**) Max-projected images of whole-mount gonads stained for HTP-3, RAD-51, and SUN-1 pS24, an indicator of activity of protein kinase CHK-2, showing comparable lengths of the ‘CHK-2 active zones’ in WT and *rad-54.B(gk340656)* gonads and illustrating that an abrupt drop in hyperaccumulated RAD-51 in the *rad-54.B* mutant coincides with the decline in CHK-2 activity that marks the transition from early to late pachytene. Scale bar represents 20 μm. Insets on the right show zoomed-in fields of nuclei from the late pachytene regions that include a few ‘outlier nuclei’ with high levels of both RAD-51 and SUN-1 pS24, reflecting the normal operation of meiotic checkpoints in the *rad-54.B* mutant. Scale bar in insets represents 5 μm. (**B**) Quantification of length of the CHK-2 active zone, defined as the contiguous region where the majority of nuclei in each cell row exhibited SUN-1 pS24 staining. Error bars represent standard deviation. Numbers of gonads analyzed: WT, *n* = 18; *rad-54.B, n* = 20. Statistical significance was assessed by Welch's t test; *P*> 0.05 (ns). (**C**) Max-projected images of RAD-51 staining in whole-mount gonads. Depicted nuclei are at the early pachytene stage for WT and *rad-54.B* (full genotype: *rad-54.B; egl-1 yIs34 oxTi633*) or from the equivalent position in the *rad-54.B; chk-2* gonad (full genotype: *rad-54.B; egl-1 chk-2*). Scale bar represents 5 μm.

**Figure 4.**
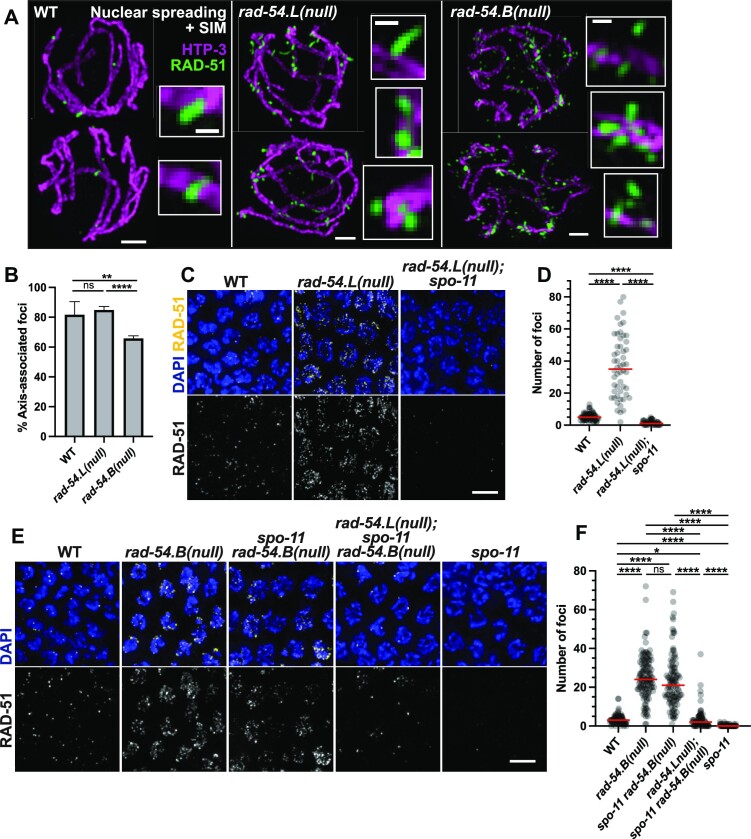
Evidence supporting RAD-51 hyperaccumulation at unbroken DNA in the *rad-54.B* mutant. (**A**) Max-projected images of RAD-51 and axis marker HTP-3 in spread early pachytene nuclei imaged by structured illumination microscopy (SIM), illustrating differences in the appearance of hyperaccumulated RAD-51 in *rad-54.L(me98)* and *rad-54.B(gk340656)* mutants (see main text). Scale bars for whole nuclei represent 1 μm; scale bars for zoomed-in insets represent 200 nm. (**B**) Graph quantifying fraction of RAD-51 foci that were scored as axis associated in SIM images such as those represented in (A). Error bars indicate 95% confidence interval. Number of RAD-51 foci assessed: WT, *n* = 60; *rad-54.L, n* = 759; *rad-54.B, n* = 2661. Statistical significance was assessed by Fisher exact test; ns, *P*> 0.05; **, *P* < 0.01; ****, *P* < 0.0001. (**C**) Representative fields of nuclei from max-projected images of whole-mount gonads, taken from the zones of maximum accumulation of RAD-51 foci; scale bar represents 5 μm. (**D**) Quantification of RAD-51 foci for the genotypes shown in (C). RAD-51 foci counts were conducted in the zone of maximum accumulation of RAD-51 foci for each genotype. Each circle represents a nucleus; red lines indicate median values. Numbers of nuclei analyzed (*n*) and median numbers of foci (*m*) were as follows: WT, *n* = 66, *m* = 5; *rad-54.L, n* = 52, *m* = 35; *rad-54.L; spo-11, n* = 49, *m* = 1. Statistical significance was assessed with a Mann–Whitney test; ****, *P* < 0.0001. (**E**) Representative fields of nuclei from max-projected images of whole-mount gonads, taken from the zones of maximum accumulation of RAD-51 foci. Scale bar represents 5 μm. (**F**) Quantification of RAD-51 foci for the genotypes shown in (E). WT, *n* = 81, *m* = 3; *rad-54.B, n* = 115, *m* = 24; *spo-11 rad-54.B, n* = 99, *m* = 21; *rad-54.L; spo-11 rad-54.B, n* = 71, *m* = 2; *spo-11, n* = 65, *m* = 0. Statistical significance was assessed with a Mann–Whitney test; ns, *P*> 0.05; *, *P* < 0.05; ****, *P* < 0.0001.

Worms were grown using standard methods (52) at 20°C, except for experiments depicted in Figure 1D, E, Figure 2, Figure 3A, B, Figure 4A, B, Figure 6D, and Supplementary Figure S2, where worms were grown at 22°C. *rad-54.B(gt3308)* was isolated in a screen for mutants with increased sensitivity to irradiation at the L1 larvae stage (53). *rad-54.B(gk340656)* was obtained from the Million Mutations Project (54). *rad-54.L(me177), rad-54.B(gt3402[rad-54.B::GFP])* and *rad-54.B(gt3328)* were generated using CRISPR-Cas9 gene editing using established methods (55–60); details are provided in Supplemental Information.

### Immunofluorescence and imaging

Immunofluorescence experiments using whole-mount gonads or spread nuclei were conducted as in (61–63); worms were dissected at 22–30 h post L4.

**Figure 5.**
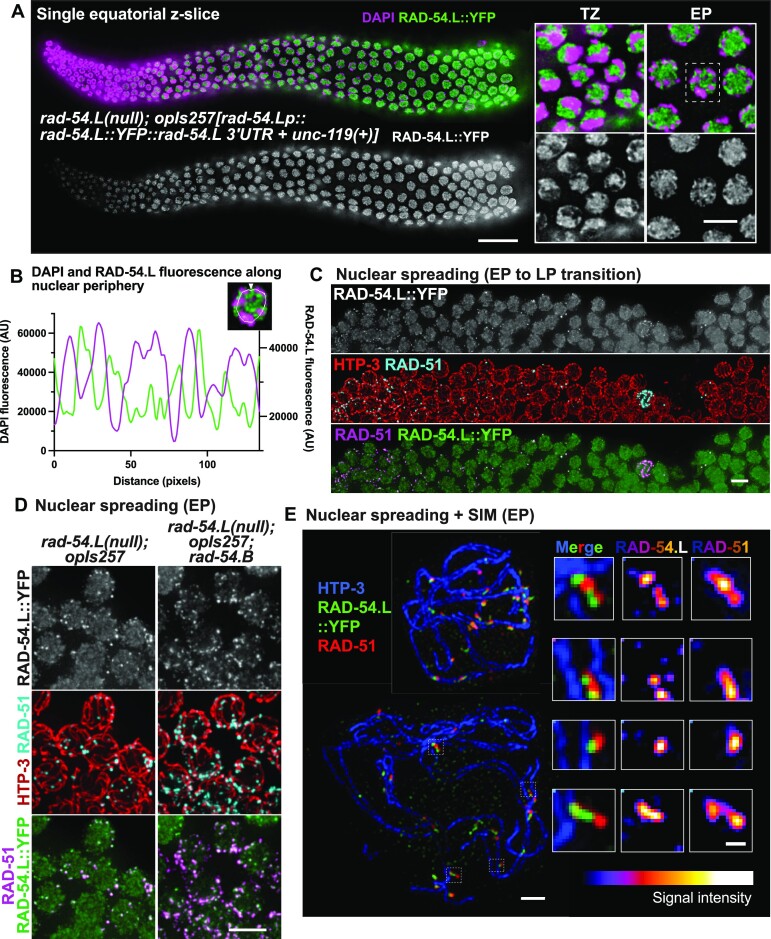
Localization of RAD-54.L. (**A**) Image of whole-mount gonad from a *rad-54.L(me98); opIs257[rad-54.Lp::rad-54.L::YFP::rad-54.L 3*′*UTR + unc-119(+)]* worm immunostained for RAD-54.L::YFP. *The image represents a single z-slice showing an equatorial view of nuclei (instead of a max-projection of whole nuclei)*. Scale bar represents 20 μm. Insets at right depict zoomed-in fields of nuclei from the transition zone (TZ) and early pachytene zone (EP), illustrating that the preponderance of RAD-54.L::YFP detected in whole-mount nuclei is not associated with the DAPI-stained chromatin. The scale bar for insets represents 5 μm. (**B**) Quantification of fluorescence levels for DAPI (magenta) and RAD-54.L::YFP (green) along the periphery of the depicted nucleus, illustrating that the RAD-54.L::YFP signal is predominantly localized in the spaces between the chromosomes, *i.e*. in the nucleoplasm; image represents a single equatorial Z-slice. Fluorescence was measured along the line indicated, starting at the arrowhead on the top and going in a counter-clockwise direction. This example nucleus is also indicated by a dotted box in (A). (**C**) Max-projected representative field of view depicting the early pachytene (EP) to late pachytene (LP) transition in a spread gonad; scale bar indicates 5 μm. Nuclear spreading reveals a subset of RAD-54.L::YFP signal localized as foci specifically in the early pachytene stage; these foci colocalize with RAD-51. (**D**) Max-projected images of immunostained spread early pachytene nuclei. The subset of RAD-54.L::YFP signals localizing as foci exhibit extensive colocalization with RAD-51 foci in both WT (*rad-54.L; opIs257*) and *rad-54.B* (*rad-54.L; rad-54.B(gk340656); opIs257*) backgrounds. Scale bar indicates 5 μm. (**E**) Max-projected early pachytene chromosome spreads imaged with SIM. Shown on the left side are two nuclei with different degrees of spreading; scale bar indicates 1 μm. Insets on the right are zoomed-in images of instances of RAD-51 and RAD-54.L::YFP colocalization from the widely-spread bottom nucleus. Fluorescence intensity of RAD-54.L and RAD-51 are depicted with an color look-up table (‘LUT Fire’ in ImageJ). Scale bar represents 200 nm.

**Figure 6.**
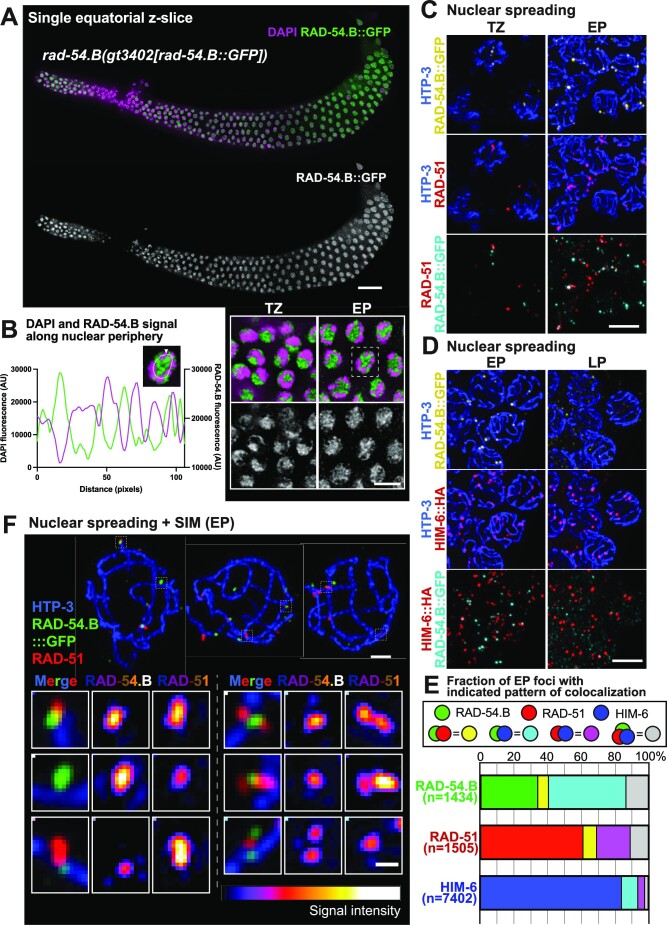
Localization of RAD-54.B. (**A**) Image of whole-mount *rad-54.B(gt3402[rad-54.B::GFP])* gonad immunostained for RAD-54.B::GFP and DAPI. *The image represents a single z-slice showing an equatorial view of nuclei (instead of a max-projection)*. Scale bar represents 20 μm. Insets at right-bottom depict a zoomed-in field of nuclei from the transition zone (TZ) and early pachytene zone (EP), showing that similarly to RAD-54.L, the preponderance of RAD-54.B is not associated with DAPI-stained chromatin. The scale bar for insets represents 5 μm. (**B**) Quantification of fluorescence levels of DAPI (magenta) and RAD-54.B::GFP (green) along the periphery of the depicted nucleus, as in Figure 5. This example nucleus is also indicated by a dotted box in (A). (**C**) Max-projected images of RAD-54.B::GFP and RAD-51 immunostaining in spread nuclei from the transition zone (TZ) and the early pachytene (EP) zone. Release of soluble RAD-54.B by nuclear spreading reveals distinct axis-associated RAD-54.B foci, a fraction of which colocalize with RAD-51 foci. Scale bar represents 5 μm. (**D**) Max-projected images of RAD-54.B::GFP and HIM-6 immunostaining in spread nuclei in early pachytene (EP) and late pachytene (LP). Distinct axis-associated RAD-54.B::GFP foci detected in early pachytene frequently colocalize with HIM-6 foci; axis-associated RAD-54.B::GFP signals diminish in late pachytene. Scale bar represents 5 μm. (**E**) Quantification of colocalization between RAD-54.B::GFP, RAD-51 and HIM-6 foci in early pachytene chromosome spreads. For each species of focus, the fractions of solo foci and foci colocalizing with one or both of the other proteins are indicated. (**F**) Max-projected early pachytene chromosome spreads imaged with SIM; scale bar represents 1 μm. Insets on bottom are zoomed-in images of instances of colocalization of RAD-51 and RAD-54.B::GFP. Fluorescence intensity of RAD-54.B and RAD-51 are depicted with an color look-up table (‘LUT Fire’ in ImageJ). Scale bar in insets represents 200 nm.

Image acquisition and processing of whole-mount gonads and spread nuclei were conducted as in (63,64). All images were acquired on a DeltaVision OMX Blaze microscope with a 100 × 1.4 numerical aperture (NA) objective, with 200 nm spaced z-stacks for widefield images and 125 nm spaced z-stacks for structured illumination microscopy (SIM) images. For widefield images, images were deconvolved and registration corrected using SoftWoRx software. Individual fields of view were assembled together for each gonad using the ‘Grid/Collection stitching’ FIJI plugin (65). For display, a single layer of nuclei was maximum-projected using FIJI, except for Figures 5A, 6A, and Supplementary Figure S8, in which a single z-slice at the center of the nuclear layer is displayed. For SIM images, images were reconstructed, registration corrected, and maximum projected using SoftWoRx software. Brightness and contrast were adjusted in FIJI for display of both widefield and SIM images.

### Chromosome tracing and quantification of CO site focus distribution

Chromosome tracing and quantification of CO site distribution (Figure 1D, Supplementary Figure S2) were done manually using the ‘Simple Neurite Tracer’ (SNT) plugin on ImageJ (66). Gonad nuclear spreads prepared from AV695 and TG4252 worms were immunostained using antibodies for HTP-3, SYP-1, GFP and MSH-5, and imaged using SIM. Nuclei in the late pachytene stage with 5 or 6 COSA-1::GFP and MSH-5 foci and with SYP-1 still localized along the entire lengths of the aligned homolog pairs were selected for quantification of CO site distribution. HTP-3 and SYP-1 were used as markers of the chromosome axis, while COSA-1::GFP and MSH-5 were used as markers for CO sites. Images were collapsed into single-channel 8-bit images and input into the ImageJ SNT plugin. For each selected nucleus, the lengths of all chromosomes were measured by manually tracing the chromosome using SNT, and the distance of each CO site to the nearest chromosome end was similarly quantified. *mnT12* fusion chromosomes and non-fusion chromosomes were distinguished after tracing by overall chromosome length, as *mnT12* chromosomes were longer than 15 μm, while all other chromosomes were shorter than 15 μm.

### Focus count quantification

Z-stack images of whole gonads were cropped to include only a single layer of germ cell nuclei. These cropped images (‘cropped Z-stacks’) were max projected, and nuclei that were well-separated from each other were manually segmented using HTP-3 signal and saved as polygon-shaped regions of interest (ROIs) in FIJI software. These ROIs were then overlayed on the cropped Z-stacks, and the area outside of the ROIs was cleared of all signal. Then, the 3D Maxima Finder plugin on ImageJ was used to identify immunofluorescence foci and determine their peak brightness and the 3-dimensional (3D) positions of their maxima (67). For 3D Maxima Finder parameters, the ‘Minimum Peak Height’ was determined empirically, starting at the max value of background fluorescence as determined by manual sampling, and iteratively running the plugin with different ‘Minimum Peak Height’ values to minimize the numbers of false positive and false negative foci identified. All other parameters (‘Radius xy’, ‘Radius z’, ‘Noise’) were kept at the default settings, 1.5, 1.5 and 100, respectively. From the 3D Maxima Finder output table of foci with their xyz peak positions and peak heights, foci were assigned to ROIs corresponding to individual nuclei based on their positions using a custom Python script. Nucleus-focus assignments were manually checked to remove overlap to generate the final foci counts. The xy position of each nucleus ROI was used to approximate its position within the region of quantification, *i.e*. the x-axis values in Figure 1E and 2B.

For whole-gonad quantifications (Figure 1E and Supplementary Figure S9C), this analysis was performed across the entire gonad. For quantification in early pachytene nuclear spreads (Figures 2B, C, 6E, Supplementary Figure S13), the early pachytene zone was defined as starting at the cell row in which most nuclei have a RAD-51 focus and ending at the cell row in which most nuclei have 6 (CO site-associated) MSH-5 or HIM-6 foci. For quantification in late pachytene nuclear spreads (Figure 2E), the late pachytene zone was defined as starting at the cell row in which most nuclei have 6 MSH-5 foci and ending before the diplotene stage when HTP-3 axis staining loses its linear appearance. For both cases, the appropriate region was cropped and used for the quantification pipeline above. For quantification of RAD-51 foci in *rad-54.L* and *rad-54.B* mutants (Figure 4D, F, and Supplementary Figure S7), a 10 cell row-wide zone corresponding to the region of peak accumulation of RAD-51 foci was manually identified and cropped before performing focus count quantification as described above. For Supplementary Figure S9F, RAD-51 foci quantification was performed in a 10 cell row-wide region corresponding to the region of peak accumulation of meiotic RAD-51 foci.

For quantification of the fraction of RAD-51 foci associated with the axis (Figure 4B), well-isolated nuclei with strong axis (HTP-3) signal were selected from SIM images and individually cropped. RAD-51 focus quantification was performed using 3D Maxima Finder as described above, twice for each nucleus: once for the whole nucleus, and once for RAD-51 signal colocalizing with the axis signal. For axis segmentation, a mask of the HTP-3 signal was generated as in (68); this mask was then dilated by 1 voxel in the z-axis to include any RAD-51 foci that were adjacent to or tethered to the axis. A Z-stack image of all RAD-51 signal within this ‘dilated axis mask’ was generated as in (68) and used to quantify the number of axis-associated RAD-51 foci. The degree of axis association for each genotype was calculated as: (Total number of axis-associated RAD-51 foci) / (Total number of RAD-51 foci).

All original 32-bit z-stack images, images cropped for analysis as described above, analysis results from 3D Maxima Finder, and Python scripts used for image analysis are available on the associated Biostudies database entry (69).

### Colocalization analysis

For colocalization analyses (Figures 2C, 6E, Supplementary Figure S9), the xyz positions and peak heights of foci were determined as outlined above, except that foci identification for Figure 6E and Supplementary Figure S9 was done without segmenting nuclei. For each species of focus quantified (*e.g*. MSH-5 in Figure 2C), the distance from each focus to the nearest neighbor focus of the other species (*e.g*. RAD-51 in Figure 2C) was determined computationally using custom Python scripts. Foci were considered colocalized if the distance to the nearest neighbor was under the appropriate threshold, calculated as the smallest resolvable distance based on the numerical aperture of the objective (1.4) and wavelength (λ) used (0.61λ/1.4, i.e. 242 nm if λ=555 nm, 282 nm if λ = 647 nm).

### 4′,6-Diamidino-2-phenylindole (DAPI) Staining of Diakinesis Oocytes

Ethanol fixation and DAPI staining of diakinesis oocytes was conducted as in (70).

### Meiotic CO distribution assay using SNP mapping strategy

Meiotic CO distribution was assayed as described (71). Additional details are available in Supplemental Information andSupplementary Table S1.

### Statistical analyses

Details on statistical tests used to evaluate significance are described for each instance in the appropriate Figure legend.

## RESULTS

### *rad-54.B* mutants undergo mostly normal meiosis but exhibit hyperaccumulation of RAD-51 foci

Our investigation of the roles of RAD-54.B was initiated by our identification of missense mutation *rad-54.B(gt3308)* (Figure 1A) in a genetic screen for *C. elegans* mutants exhibiting an increased sensitivity to ionizing radiation at the L1 larval stage (53). Given that the *C. elegans* RAD-54.B paralog RAD-54.L (previously known as RAD-54) is essential for DSBR during meiotic recombination, and that the *S. cerevisiae* RAD-54.B ortholog Rdh54/Tid1 contributes to meiotic recombination in budding yeast, we investigated potential effects of loss of *rad-54.B* function on progression and success of meiotic recombination.

Several lines of evidence indicate that RAD-54.B is largely dispensable for successful meiosis in *C. elegans*. First, worms homozygous either for *rad-54.B(gt3308)* or for putative null allele *rad-54.B(gk340656)* exhibit nearly wild type (WT)-like levels of embryonic viability and male progeny (Figure 1A, B); this contrasts with expectations for *C. elegans* mutants defective in meiotic recombination, which exhibit a high rate of embryonic lethality (reflecting failure of DSBR or missegregation of autosomes) and/or a ‘high incidence of male’ progeny (the Him phenotype, reflecting sex-chromosome mis-segregation). We also quantified COSA-1 foci, which mark crossover (CO) sites at the late pachytene stage of meiotic prophase. Six COSA-1 foci are typically observed in WT nuclei, corresponding to a single CO site for every chromosome pair (47). Six COSA-1 foci per nucleus were similarly detected in *rad-54.B(gk340656)* late-pachytene meiocytes, suggesting that COs are specified in normal numbers in this mutant (Figure 1C). Further, distributions of the positions of COSA-1 foci along chromosome axes were similar in control and mutant meiocytes (Figure 1D), and fusion-chromosome assays for CO patterning likewise did not detect differences between mutant and control (Supplementary Figure S2). Together, these data suggest that the mechanisms required to initiate meiotic recombination and to promote and regulate the designation of meiotic CO sites are largely operational in *rad-54.B* mutants.

Despite the above evidence for substantially successful CO formation and meiotic chromosome segregation in *rad-54*.B mutants, immunostaining for recombinase RAD-51 revealed a striking temporary hyperaccumulation of RAD-51 foci in two independently-derived *rad-54*.*B* presumed null mutants, *rad-54.B(gk340656)* and *rad-54.B(gt3328)* (Figure 1E, Supplementary Figure S1). In a WT whole-mount gonad, RAD-51 can be visualized as chromosome-associated foci in prophase nuclei starting at the transition zone and ending midway through pachytene (Figure 1E, Supplementary Figure S1). In contrast, both *rad-54.B* mutants exhibit hyperaccumulation of RAD-51 foci within a specific region of the germ line, starting at meiotic prophase onset and declining abruptly midway through prophase I (Figure 1E, Supplementary Figure S1). In most subsequent experiments in this study, we use *rad-54.B(gk340656)*, a presumed null allele, unless otherwise specified.

We note that the temporary hyperaccumulation of RAD-51 observed in *rad-54.B* mutants contrast sharply with RAD-51 hyperaccumulation observed in *rad-54.L* mutants, in which RAD-51 foci continue to accumulate throughout meiotic progression (Supplementary Figure S1). Given this difference in RAD-51 hyperaccumulation, we further investigated the progression of meiotic DSBR in these mutants.

### Meiotic DSBR progression is severely impaired in *rad-54.L* mutant but appears substantially normal in *rad-54.B* mutant

We investigated progression of meiotic DSBR in *rad-54.B* and *rad-54.L* mutants by simultaneous immunostaining for RAD-51 and MSH-5 in spread preparations that improve detection of chromosome-associated recombination proteins. MSH-5, which partners with MSH-4 to comprise the MutSγ complex (72), is a meiosis-specific DSBR factor that initially localizes to numerous interhomolog recombination intermediates in early pachytene nuclei and becomes concentrated specifically at CO-designated sites upon transition to late pachytene (47,63). Time course analysis indicates that early pachytene MSH-5 foci in WT meiosis represent post-strand-exchange intermediates (63).

Our cytological analysis revealed several notable differences between the *rad-54.L* and *rad-54.B* mutants. First, abundant MSH-5 foci accumulate in *rad-54.B* early pachytene nuclei, albeit with an apparent delay (Figure 2A, B). In contrast, numbers of MSH-5 foci in the *rad-54.L* mutant are greatly reduced relative to either WT or *rad-54.B* (Figure 2A, B). Second, while MSH-5 foci and RAD-51 foci rarely colocalize in either WT or *rad-54.B* early pachytene nuclei, despite increased numbers of RAD-51 foci in the *rad-54.B* mutant, the few MSH-5 foci observed in *rad-54.L* early pachytene nuclei frequently colocalize with RAD-51 (Figure 2A, C). Together, these observations suggest that in the *rad-54.L* mutant, the inability to remove RAD-51 from DSBR sites prevents the stable association of subsequent factors such as MSH-5 to DSBR sites. However, in the *rad-54.B* mutant, the early steps of meiotic DSBR progress relatively normally.

Consistent with the successful progression of meiotic DSBR and designation and formation of CO recombination intermediates, MSH-5 foci counts in late pachytene spreads from the *rad-54.B* mutant were largely comparable to those observed in WT (WT, 5.9 ± 0.5; *rad-54.B*, 5.9 ± 0.8; Mann–Whitney *p* = 0.66), although a higher incidence of nuclei with fewer or greater than 6 MSH-5 foci suggests that regulation of CO-factor accumulation my be less robust in the *rad-54.B* mutant (Figure 2D, E). In contrast to the *rad-54.B* mutant, late pachytene nuclei in the *rad-54.L* mutant retain abundant hyperaccumulated RAD-51 foci and exhibit substantially reduced numbers of MSH-5 foci (1.9 ± 1.0) (Figure 2D, E). These findings further support the conclusion that *rad-54.B* mutants are largely proficient for progression of meiotic DSBR, while *rad-54.L* mutants are severely compromised for progression of DSBR beyond the formation of early RAD-51 bound intermediates.

### RAD-51 hyperaccumulation in *rad-54.B* mutants is regulated by CHK-2 activity

As hyperaccumulation of RAD-51 foci in *rad-54.B* mutants is confined to a limited region of the germ line similar to where RAD-51 foci are observed in WT germ lines, we investigated the relationship of this hyperaccumulation phenotype to the activity of protein kinase CHK-2, a key regulator of multiple events in *C. elegans* meiosis.

CHK-2 activity is turned on at meiotic prophase onset, and it is essential for nuclear reorganization at meiotic entry, homolog pairing, normal SC assembly, and the formation of programmed meiotic DSBs (49,73–76). Multiple steps in meiotic recombination occur during the period of prophase when CHK-2 is active: DSBs are formed and processed (49,76), RAD-51 is loaded onto processed DNA ends to promote homolog engagement and strand exchange (48), and early RAD-51-associated recombination intermediates are converted into post-strand exchange interhomolog intermediates that accumulate throughout the early pachytene stage (63). Further, CHK-2 activity is turned off at the transition from early to late pachytene, when the requirements for the ‘CO assurance checkpoint’—that every chromosome has a DSBR intermediate that is eligible to be differentiated into a CO—are fulfilled, as evidenced by the observation of an extended ‘CHK-2 active zone’ in mutants that fail to form CO intermediates (49,73,76,77). At this transition from early to late pachytene, several key changes occur in the meiotic program, such as the shutting down of the DSB-induction machinery, changes in the molecular requirements for DSBR, and a switch in the preferred DSBR repair template from homologous chromosome to sister chromatid (49,76,78,79).

Visualization of SUN-1 pS24 as a marker of CHK-2 activity (50,77) allowed us to make several inferences regarding RAD-51 hyperaccumulation and meiotic prophase progression in the *rad-54.B* mutant. First, co-staining for SUN-1 pS24 and RAD-51 revealed a striking temporal/spatial correspondence between the abrupt decline of RAD-51 hyperaccumulation and the end of the CHK-2 active zone (Figure 3A). The rapid disappearance of RAD-51 immediately follows the shut-down of CHK-2 activity; this suggests that hyperaccumulation of RAD-51 in the *rad-54.B* mutant may depend on CHK-2 activity. Further, ‘outlier’ nuclei with high RAD-51 signal in the late pachytene region of the gonad also consistently exhibited SUN-1 pS24 signal, strengthening the inference that CHK-2 activity state and RAD-51 hyperaccumulation are linked. Second, quantification of the length of the CHK-2 active zone did not reveal any difference between WT and the *rad-54.B* mutant (Figure 3A, B), indicating apparently normal timing of progression from early pachytene to late pachytene in the *rad-54.B* mutant. This implies that the requirements of the CO assurance checkpoint are being satisfied in a timely manner, consistent with our observations regarding successful progression of meiotic DSBR.

Consistent with our hypothesis that CHK-2 activity is necessary for the hyperaccumulation of RAD-51 in the *rad-54.B* mutant, we found that RAD-51 hyperaccumulation was abrogated in a *rad-54.B; chk-2* double mutant (Figure 3C, Supplementary Figure S3). As *chk-2* mutants lack meiotic DSBs, however, this experiment did not distinguish whether RAD-51 hyperaccumulation is dependent on CHK-2 activity *per se* or on DSB formation; this issue will be addressed below.

### RAD-51 accumulates at DSBR sites in *rad-54.L* mutant germ cells but accumulates on unbroken DNA in the *rad-54.B* mutant

The observed differences in the dynamics of accumulation of RAD-51 foci and progression of DSBR in *rad-54.L* and *rad-54.B* germ lines raised the possibility that the way in which hyperaccumulated RAD-51 associates with chromosomes might be fundamentally different between these mutants. To address this possibility, we used structured illumination microscopy (SIM) to examine the morphology and spatial organization of RAD-51 foci in spread nuclei immunostained for RAD-51 (Figure 4A). SIM imaging of early pachytene nuclear spreads showed that while *rad-54.L* nuclei have an elevated number of RAD-51 foci, the RAD-51 foci have several similarities to those seen in WT. First, similarly to meiotic DSBR foci in WT meiosis (63), RAD-51 foci in the *rad-54.L* mutant are usually associated with the chromosome axes (Figure 4B). Second, in WT nuclei, RAD-51 foci are often observed as elongated foci or doublets, which are interpreted to represent RAD-51 localizing on both resected ends of a DSB (63); RAD-51 foci in the *rad-54.L* mutant are similarly observed as extended singlets or doublets, with some foci exhibiting hyper-elongation. In *rad-54.B* nuclei, in contrast, RAD-51 foci are often not associated with the chromosome axis, and foci exhibit more variability in morphology (Figure 4A, B). These data, together with the differences in DSBR progression in *rad-54.L* and *rad-54.B* mutants, suggested that the hyperaccumulated RAD-51 in these two *rad-54* family mutants might represent RAD-51 localizing at different underlying DNA structures.

Consistent with this hypothesis, we found that the *rad-54.L* and *rad-54.B* mutants differ regarding whether hyperaccumulation of RAD-51 is dependent on the formation of meiotic DSBs. We confirmed previous work showing that hyperaccumulation of RAD-51 foci is abolished in a *rad-54.L; spo-11* double mutant, which lacks the enzyme responsible for generating meiotic DSBs (28,80) (Figure 4C, D, Supplementary Figure S4A). This indicates that RAD-51 hyperaccumulation in the absence of RAD-54.L is DSB-dependent and likely occurs at meiotic DSBR sites. In contrast, hyperaccumulation of RAD-51 foci in the absence of RAD-54.B occurs independently of meiotic DSBs, as hyperaccumulation persisted in the *spo-11 rad-54.B* double mutant (Figure 4E, F, Supplementary Figure S4B). We infer that loss of *rad-54.B* function results in hyperaccumulation of RAD-51 on unbroken DNA, similarly to the previously-described DSB-independent accumulation of meiotic recombinase Dmc1 in *S. cerevisiae* mutants lacking the RAD-54.B ortholog Rdh54/Tid1 (45). We note that the zone of RAD-51 hyperaccumulation in the *spo-11 rad-54.B* double mutant is extended compared to the *rad-54.B* single mutant (Supplementary Figures S4B, S5A), which can be partially attributed to triggering of the previously-described CO-assurance checkpoint (49,76,77) in the absence of SPO-11 and resulting extension of the CHK-2 active zone (Supplementary Figure S5B).

The striking difference between the *rad-54.L* and *rad-54.B* mutants regarding DSB-dependence/independence of RAD-51 hyperaccumulation dovetails with our data showing that meiotic DSBR is stalled at an early intermediate in the *rad-54.L* mutant, but progresses successfully in the *rad-54.B* mutant. Together these findings indicate a major role for RAD-54.L in promoting progression of meiotic DSBR through a mechanism involving the removal of RAD-51 from early recombination intermediates, and a distinct role for RAD-54.B in preventing the accumulation of RAD-51 on unbroken DNA. Further, the demonstration of DSB-independent RAD-51 hyperaccumulation in the *spo-11 rad-54.B* double mutant indicates that loss of hyperaccumulation in the *rad-54.B; chk-*2 double mutant is not due to the lack of programmed DSBs, but instead reflects a role for CHK-2 in enabling association of RAD-51 with unbroken DNA when RAD-54.B is absent (Supplementary Figure S3).

### Counterbalancing effects of RAD-54.L and RAD-54.B in modulating association of RAD-51 with unbroken DNA

Having uncovered a role for RAD-54.B in antagonizing the accumulation of RAD-51 on unbroken DNA in meiotic prophase germ cells, we investigated the potential involvement of RAD-54.L in the hyperaccumulation phenomenon. Surprisingly, examination of RAD-51 localization in a *rad-54.L; spo-11 rad-54.B* triple mutant revealed that RAD-51 hyperaccumulation was greatly attenuated compared with the *spo-11 rad-54.B* double mutant (Figure 4D, E, Supplementary Figure S4B). Specifically, while *rad-54.L; spo-11 rad-54.B* triple mutant germ lines had a few ‘outlier’ nuclei with high levels of RAD-51 foci, the majority of nuclei had zero or only one or two RAD-51 foci with peak intensities above a baseline threshold for confident focus calling. Moreover, the 1–2 bright foci detected in most meiotic prophase nuclei likely represent the persistence of RAD-51 accumulated at sites of DNA damage incurred during DNA replication in the absence of both RAD-54.L and RAD-54.B, as such foci are also present in the mitotic proliferation zone at the distal end of the germ line in both *rad-54.L; rad-54.B* and *rad-54.L; spo-11 rad-54.B* mutant gonads (Supplementary Figure S4). The unexpected requirement for RAD-54.L to achieve DSB-independent RAD-51 hyperaccumulation suggests that in the absence of RAD-54.B, RAD-54.L promotes promiscuous binding of RAD-51 to unbroken dsDNA.

To further investigate the contribution of RAD-54.L to promoting DSB-independent RAD-51 hyperaccumulation, we created a *rad-54.L* missense allele (*rad-54.L(me177)*, referred to as *rad-54.L(K238R)*) encoding a predicted ATPase-dead version of the RAD-54.L protein (81,82). *rad-54.L(K238R)* single mutant gonads exhibit persistent hyperaccumulation of RAD-51 comparable to that observed in the *rad-54.L(me98)* null mutant (hereafter referred to as *rad-54.L(null)*) (Supplementary Figure S6), consistent with the ATP-dependent motor activity of RAD-54.L being required to promote removal of RAD-51 at meiotic DSBR sites. Immunostaining of *rad-54.L(K238R); spo-11 rad-54.B* germ lines (Supplementary Figures S7, S8) further revealed a substantial attenuation of SPO-11-independent RAD-51 hyperaccumulation relative to *spo-11 rad-54.B*. However, the residual RAD-51 immunostaining observed in *rad-54.L(K238R); spo-11 rad-54.B* germ lines was also distinguishable from that observed in *rad-54.L(null); spo-11 rad-54.B* germ lines; whereas 1–2 bright RAD-51 foci were detected in most nuclei for both genotypes (presumably reflecting persistence of replication-associated DNA damage as discussed above), additional residual foci were more abundant in *rad-54.L(K238R); spo-11 rad-54.B* germ lines. This intermediate abundance of RAD-51 foci in the ATPase-dead mutant background suggests that RAD-54.L may promote the association of RAD-51 with unbroken DNA through both ATPase-dependent and ATPase-independent mechanisms.

We also created *rad-54.B(gt3379)*, referred to as *rad-54.B(K244A)*, a missense allele modeled after the demonstrated ATPase-deficient mutant of *S. cerevisiae rdh54/tid1* by altering the homologous conserved residue in the Walker A motif (83). Similarly to the *rad-54.B(null)* mutant, *rad-54.B(K244A)* mutants successfully undergo meiotic prophase (Supplementary Figure S9A). As the ATPase activity of *S. cerevisiae* Rdh54/Tid1 is required to prevent accumulation of meiotic recombinase Dmc1 on unbroken DNA during yeast meiosis (45), we had anticipated that the ATPase activity of RAD-54.B would similarly be required to prevent accumulation of RAD-51 on unbroken DNA in *C. elegans* meiocytes. Counter to our expectations, meiotic prophase RAD-51 foci in the *rad-54.B(K244A)* mutant were detected at levels comparable to WT and were *spo-11-*dependent, implying that RAD-54.B ATPase activity is not required to antagonize DSB-independent RAD-51 hyperaccumulation on meiotic prophase chromosomes (Supplementary Figures S9B, C and S9E, F). A possible explanation for this unexpected observation will be discussed below.

Although meiotic RAD-51 foci were not elevated in the *rad-54.B(K244A)* mutant, this mutant did exhibit an elevation of RAD-51 foci in nuclei in the mitotic proliferation zone that disappeared around entry into meiotic prophase, as marked by CHK-2-dependent phosphorylation of nuclear envelope protein SUN-1 (Supplementary Figure S9D). This observation implies that the ATPase activity of RAD-54.B likely is relevant for RAD-51 removal during mitotic cell cycles.

### Extensive colocalization of RAD-54.L with both normal and hyperaccumulated RAD-51 foci

To better understand the roles of RAD-54.L and RAD-54.B, we investigated their localization in meiotic prophase germ cells. We assayed RAD-54.L localization by immunostaining for RAD-54.L::YFP in the germ lines of worms expressing a functional *rad-54.L::YFP* transgene in a *rad-54.L* null background (84). In whole-mount gonad preparations, RAD-54.L::YFP immunostaining was detected predominantly in the nucleoplasm (and variably in the nucleolus) (Figure 5A, B, Supplementary Figure S10-S11). Thus, to visualize potential chromosome-associated RAD-54.L::YFP signals, we used a nuclear spread preparation to release nucleoplasmic protein pools (Figure 5C, D). Nuclear spreading revealed a subset of RAD-54.L::YFP localizing as foci (*i.e*. bright signals that emerge above the general nuclear signal) in early pachytene nuclei, and these foci exhibited nearly complete colocalization with RAD-51 foci, in both WT and *rad-54.B* mutant backgrounds (Figure 5C, D). This suggests that RAD-54.L colocalizes with chromosome-associated RAD-51 regardless of whether RAD-51 is bound at DSBR sites or at unbroken DNA. Further, SIM imaging of these RAD-54.L::YFP foci showed that RAD-54.L::YFP foci are associated with chromosome axes and often occur as doublets or elongated singlets, similar to and colocalizing with RAD-51 (Figure 5E). In highly spread nuclei, partial spatial separation of RAD-51 and RAD-54.L::YFP signals became evident (Figure 5E, bottom), suggesting that RAD-51 and RAD-54.L associate with adjacent but non-identical portions of the underlying DNA molecules. However, we could not discern a stereotyped configuration of RAD-54.L::YFP relative to RAD-51 at these sites, as a variety of colocalization patterns were observed.

### Localization of RAD-54.B at a subset of meiotic DSBR sites

To visualize RAD-54.B, we used CRISPR-Cas9 genome editing to generate a strain expressing RAD-54.B::GFP from the endogenous *rad-54.B* locus. In whole-mount gonads, RAD-54.B::GFP was detected predominantly in the nucleoplasm and the nucleolus, as observed for RAD-54.L::YFP (Figure 6A, B, Supplementary Figure S12). Further, nuclear spreading likewise revealed a subpopulation of RAD-54.B::GFP in pachytene nuclei localizing to foci associated with the chromosome axis (Figure 6C, D). While a subset of RAD-54.B::GFP foci colocalized with RAD-51 foci, however, the majority did not; conversely, the majority of RAD-51 foci did not colocalize with RAD-54.B::GFP foci (Figure 6C, E). This contrasts sharply with the essentially complete coincidence observed for RAD-51 and RAD-54.L::YFP foci (above), presumably reflecting distinct roles and contributions of RAD-54.L and RAD-54.B.

Examination of sites of RAD-51 and RAD-54.B::GFP colocalization by SIM imaging (Figure 6F) revealed overlapping or adjacent but non-identical localization of the two proteins at these sites, but no discernable stereotypical spatial relationship was evident. We observed cases of a RAD-51 singlet adjacent to a RAD-54.B::GFP singlet, as well as cases in which a RAD-51 doublet flanks RAD-54.B::GFP, or vice versa.

We further assessed the relationship of RAD-54.B::GFP foci to meiotic DSBR sites by costaining for HIM-6::HA (BLM helicase). This analysis revealed: a) partial colocalization between RAD-54.B::GFP foci and HIM-6::HA foci in early pachytene nuclei, and b) lost or diminished axis-associated RAD-54.B::GFP signal in late pachytene nuclei, in which HIM-6::HA localization is restricted to a single CO-designated site per chromosome pair (Figure 6D, E).

We quantified colocalization between RAD-54.B::GFP, RAD-51, and HIM-6::HA in early pachytene spread nuclei co-stained for all 3 proteins (Figure 6E). For each protein, we quantified the fraction of foci that were observed alone or colocalizing with one or both other proteins. This analysis indicated that: a) the majority of RAD-54.B::GFP foci colocalize with RAD-51 (92/1434, 6.4%), HIM-6::HA (660/1434, 46.0%), or both (195/1434, 13.6%), but b) only a minority of RAD-51 foci (287/1505, 19.1%) or HIM-6 foci (889/7402, 12.0%) colocalize with RAD-54.B::GFP (Figure 6E). Colocalization of RAD-54.B foci with foci marking meiotic DSBR sites suggests that in addition to its role in antagonizing accumulation of RAD-51 on unbroken DNA, RAD-54.B may also play a role (albeit non-essential) in meiotic DSB repair.

In addition to the colocalization analysis, the preservation of temporal/spatial organization of nuclei in the germ line in the imaged samples allowed us to plot the positions of the different classes of foci (categorized by colocalization pattern) along an axis corresponding to temporal progression through the early pachytene stage (Supplementary Figure S13A). While all types of foci were present throughout most of this spatial ‘time course’, analysis of relative distributions and median positions showed that RAD-51 foci tend to appear earlier than RAD-54.B::GFP foci, which in turn tend to precede HIM-6::HA foci. Further, HIM-6::HA foci that colocalize with RAD-51 (with or without RAD-54.B::GFP) tend to precede HIM-6::HA foci that colocalize only with RAD-54.B::GFP, and these further tend to precede HIM-6::HA foci that are observed on their own. Our combined colocalization and temporal analyses suggest that RAD-54.B may associate transiently with DSBR intermediates during their transition from early intermediates marked by RAD-51 alone into later post-strand-exchange intermediates marked by HIM-6 alone.

Although the majority of RAD-54.B::GFP foci colocalized with RAD-51 and/or HIM-6::HA foci, suggesting participation in DSBR, 487/1434 (34%) of RAD-54.B::GFP foci did not colocalize with either. These solo RAD-54.B::GFP foci tended to be dimmer and appear later than RAD-54.B::GFP foci colocalizing with RAD-51 and/or HIM-6::HA (Supplementary Figure S13A, B).

### Evidence that RAD-54.B makes non-essential contributions to meiotic DSBR

Although our initial analyses had demonstrated that RAD-54.B is largely dispensable for successful meiosis, localization of RAD-54.B in foci at DSBR sites during meiotic prophase raised the possibility that RAD-54.B might nevertheless contribute in a non-essential way to meiotic DSB repair. Thus, we sought additional evidence for participation of RAD-54.B in meiotic DSBR.

We first used a genetic assay to assess the distribution of COs relative to a set of Chromosome V SNP markers in control and *rad-54.B* mutant backgrounds (Supplementary Figure S14). Consistent with our cytological observation of 6 COSA-1 foci in late pachytene nuclei in *rad-54.B* mutants, we did not detect a reproducible difference between control and the *rad-54*.B mutant in the total frequency of Chromosome V COs. However, data from two independent experiments did suggest that the distribution of COs among intervals is modestly altered in a *rad-54.B* mutant background (Expt. 1, *P* = 0.03; Expt. 2, *P* = 0.003).

More definitive evidence for a contribution of RAD-54.B to meiotic DSBR was provided by experiments using *rad-54.L(me139)*, a partial loss-of-function mutant (85), as a sensitized genetic background. Assessment of the viability of embryos produced by WT, *rad-54.B* and *rad-54.L(me139)* single mutants, and the *rad-54.L(me139); rad-54.B* double mutant revealed that loss of RAD-54.B in the *rad-54.L(me139)* mutant background resulted in a substantial decrease in embryonic viability (Table 1). In contrast to the 98% and 26% egg hatching rates observed for the *rad-54.B* and *rad-54.L(me139)* single mutants, respectively, the *rad-54.L(me139); rad-54.B* double mutant exhibited 100% embryonic lethality (0% egg hatching), similarly to a *rad-54.L* null mutant.

**Table 1.** RAD-54.B can contribute to successful reproduction and development in a *rad-54.L* partial loss-of-function background

Genotype	Total number of eggs	% Eggs hatching	% Eggs reaching adulthood	% Males (/adults)
WT	869	99.9	99.9	0
*rad-54.B(gk340656)*	996	98.2	97.9	0.2
*rad-54.L(me139)* ^a^	961	26.3	15.2	15.8
*rad-54.L(me139); rad-54.B(gk340656)*	777	0	0	NA

For progeny viability counts, single L4 hermaphrodites (4 for each genotype) were placed on individual plates, then each worm was transferred to a new plate at 24 h intervals until 72 h post L4. Numbers of eggs and larvae were counted 24 h (time point 1) and 48 h (time point 2) after plating of the parent worm; numbers of adult hermaphrodites and males were counted 72–96 hours after plating (time point 3). ‘Total number of eggs’ laid was taken to be the sum of eggs and larvae at either time point 1 or 2, or the total number of adult worms at time point 3, whichever was larger, to account for both undercounting of young larvae and larval lethality. % Eggs hatching = 100 × (1 – unhatched eggs at time point 2/total eggs laid).

^a^For *rad-54.L(me139)*, data are from reference (85). The difference between ‘% Eggs hatching’ and ‘% Eggs reaching adulthood’ reflects larval arrest/lethality.

**Figure 7.**
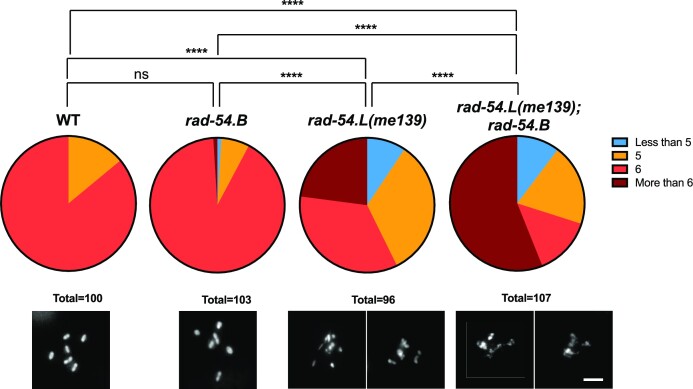
Evidence that RAD-54.B contributes to meiotic DSBR. Top, quantification of the fraction of oocytes displaying the indicated numbers of DAPI-stained bodies. Bottom, max-projected images of DAPI-stained diakinesis oocytes for each genotype. *rad-54.L(me139); rad-54.B(gk340656)* oocytes had more irregularities in DAPI body morphology than *rad-54.L(me139)* oocytes, often appearing ‘stringy’ and exhibiting more variability in size. While some *rad-54.L(me139)* and *rad-54.L(me139); rad-54.B* oocytes were scored as having 5–6 DAPI bodies, in many cases these did not represent WT-like bivalents, but instead may represent products of failed DSB repair such as chromosome fragments or fused chromosomes. Although DAPI-body number is an imperfect representation of such abnormal diakinesis figures, this quantification does capture the fact that the defective diakinesis phenotype caused by *rad-54.L(me139)* is aggravated by simultaneous loss of *rad-54.B*. Scale bar represents 5 μm. Fisher exact tests were used to compare genotypes for the fraction of oocyte nuclei exhibiting abnormal diakinesis figures (i.e. <5 or > 6 DAPI bodies): for WT vs *rad-54.B, p*> 0.05 = ns; for all other pairwise comparisons, *P* < 0.0001.

Further, assaying chromosome morphology in diakinesis oocytes by DAPI staining revealed that loss of *rad-54.B* function aggravates the diakinesis defects observed in the *rad-54.L(me139)* mutant background (Figure 7). Six bivalents are usually observed in WT and *rad-54.B* diakinesis oocytes, reflecting successful formation of CO-based attachments between homologs and completion of DNA repair during earlier stages of prophase. In contrast, DAPI-stained bodies in diakinesis oocytes in the *rad-54.L(me139)* mutant exhibit more variability in size and shape, a phenotype indicative of a defect in DSBR, which may cause chromosome fragmentation and/or inappropriate associations (48,86). This abnormal diakinesis phenotype was aggravated in the *rad-54.L(me139); rad-54.B* double mutant, where DAPI-stained bodies in diakinesis oocytes were also highly variable in size and shape and deviated even further from normal-appearing bivalents, frequently exhibiting a ‘stringy’ appearance in which many DAPI bodies appeared to be connected by thin DAPI-stained threads or bridges. Further, the fraction of oocyte nuclei exhibiting abnormal phenotypes (*i.e*. categorized as having fewer than 5 or greater than 6 DAPI bodies) was significantly higher in the *rad-54.L(me139); rad-54.B* double mutant than in the *rad-54.L(me139)* single mutant. Taken together, these data reveal a hidden capacity of RAD-54.B to contribute to the successful completion of meiotic DSBR.

## DISCUSSION

Our current work clearly shows that RAD-54.L and RAD-54.B make very different contributions to the success of homologous recombination in the *C. elegans* germ line. We demonstrate a division of labor between and counterbalancing effects of these RAD-54 paralogs during *C. elegans* meiosis, with RAD-54.L in the crucial leading role and RAD-54.B as a supporting player.

We confirm prior work indicating an essential role for RAD-54.L in promoting the removal of RAD-51 from meiotic DSBR sites, and we extend this finding to demonstrate a requirement for a functional ATPase domain, implying that the translocase activity of RAD-54.L is important for its role in displacement of RAD-51. We further show that RAD-54.L is required for normal progression of subsequent steps in meiotic DSBR, including recruitment of the MutSγ complex, consistent with RAD-51 removal and MutSγ recruitment being mechanistically coupled events. Moreover, in the *rad-54.L* mutant background, RAD-51 colocalizes with MutSγ at the small subset of DSBR sites where MutSγ is recruited, reflecting impairment in the transition from recombinase-bound intermediates to post strand-exchange intermediates lacking RAD-51.

The strong mechanistic connections between RAD-51 and RAD-54.L are further reflected in their extensive colocalization, which suggests that RAD-51 filaments rarely occur without RAD-54.L being in close proximity, and conversely, that RAD-54.L rarely occurs in foci without RAD-51. RAD-54.L foci colocalized with RAD-51 foci may represent RAD-54.L translocases actively promoting homology search by mediating motor-driven one-dimensional movement of RAD-51-ssDNA filaments (21) and/or they may represent RAD-54.L translocases engaged in the process of RAD-51 removal, thereby driving strand exchange and D-loop formation (12,87). Alternatively, or in addition, colocalized foci may reflect a RAD-51-filament-stabilizing activity of RAD-54.L (see below).

In striking contrast to the central and essential role for RAD-54.L in meiotic DSBR, we found that RAD-54.B is largely dispensable for successful meiotic recombination. This indicates that despite *C. elegans* having only a single RAD-51 recombinase, the two RAD-54 paralogs must partner with RAD-51 in very different ways; further, RAD-54.B cannot substitute for RAD-54.L to complete the essential tasks of removing RAD-51 and enabling DSBR progression. However, localization of RAD-54.B at a minority of meiotic DSBR sites during wild-type meiosis and a modest delay in the appearance of MSH-5 foci in the *rad-54.B* null mutant background together suggest that RAD-54.B likely does play a transient, albeit largely non-essential, role during meiotic DSBR. Based on prior studies identifying several disparate biochemical activities for RAD54 family translocases, we speculate that RAD-54.B might potentially function in multiple capacities at DSBR sites. For example, it might affect the structure of DSBR intermediates by modulating D-loop size or D-loop maturation, as proposed for yeast Rdh54/Tid1 (39,40), and/or it might modulate the activity of RAD-54.L in promoting RAD-51 removal. RAD-54.B functioning in such capacities at DSBR sites may underlie the modest difference in CO distribution observed in the *rad-54.B* mutant. Further, while RAD-54.B cannot substitute for RAD-54.L, it may play an auxiliary role in removing RAD-51 from DSBR sites, which may explain the contribution of RAD-54.B to meiotic success in a *rad-54.L* partial loss-of-function background. We further speculate that such a role for RAD-54.B in removing RAD-51 from DSBR sites may be more important during mitotic cell cycles than during meiosis, based on detection of elevated RAD-51 foci in mitotically-cycling and meiotic S-phase germ cells, but not meiotic prophase germ cells, in the ATPase-dead *rad-54.B(K244A)* mutant.

The most conspicuous function uncovered for RAD-54.B in this work is its role in preventing hyperaccumulation of RAD-51 on unbroken DNA. This finding parallels previous observations that the RAD-54.B ortholog Rdh54/Tid1 is required to inhibit the association of meiotic recombinase Dmc1 with unbroken DNA during *S. cerevisiae* meiosis (45) and that both RAD-54.B and RAD-54.L orthologs contribute to antagonizing unproductive RAD51 accumulation and associated toxicity when RAD51 is overexpressed in vegetative yeast cells or cancer cells (43,44). However, the activity of budding yeast Rdh54 in antagonizing off-target recombinase accumulation was shown to be dependent on its ATPase activity, suggesting that recombinase complexes associated with undamaged dsDNA are removed by Rdh54 proteins translocating along DNA (44,45). In contrast, our analysis of the ATPase-dead *rad-54.B(K244A)* missense mutant revealed that *C. elegans* RAD-54.B antagonizes hyperaccumulation of RAD-51 on unbroken DNA in an ATPase-independent manner. This surprising finding indicates that *C. elegans* RAD-54.B must prevent RAD-51 from becoming concentrated at off-target foci by an alternative mechanism that does not involve translocase-mediated removal of RAD-51 from dsDNA-associated filaments.

How might RAD-54.B function in an ATPase-independent manner to inhibit RAD-51 accumulation on unbroken DNA? Given that the vast majority of RAD-54.B in *C*. elegans germ cells is localized in the nucleoplasm, we propose that rather than RAD-54.B actively removing RAD-51 from DNA, the soluble nucleoplasmic pool of RAD-54.B may function to bind and sequester RAD-51 protomers in the nucleoplasm, thereby limiting their availability to form unproductive filaments on unbroken DNA. We further hypothesize that the RAD-54.B N-terminal domain may play a role in this function. The N-terminal domains (NTDs) of RAD54L/Rad54 and RAD54B/Rdh54 have diverged substantially since the ancient duplication that gave rise to the two paralogs, and for budding yeast Rad54 and Rdh54, this N-terminal divergence has been demonstrated to contribute to the divergence in their roles and activities *in vivo* and *in vitro* (41). Further, Rdh54 can bind to Rad51 *in vitro* in the absence of DNA, and its ability to do so depends on its NTD, but not its ATPase activity or its translocase domain (88). We note that the largely unstructured NTDs of RAD54B orthologs harbor a conserved 70–80 amino acid structured domain predicted by AlphaFold (89) that is absent from RAD54L orthologs. We propose that this RAD-54.B-specific N-terminal structured motif may bind RAD-51 and help sequester it in the nucleoplasm to prevent its promiscuous binding to unbroken DNA.

Affinity of both eukaryotic and prokaryotic recombinases for dsDNA has been proposed to be an inherent and essential feature of these proteins, as recombinase filaments become associated with the hybrid dsDNA product of a successful homology search and strand-exchange reaction during the normal course of recombination (90). Thus, our observation that *C. elegans* RAD-51 exhibits a similar propensity for promiscuous association with unbroken DNA in the absence of RAD-54.B is consistent with the idea that this property is a conserved aspect of recombinases. The authors of the cited review further proposed that ATP expenditure, either by the recombinase itself in the case of bacterial RecA, or by RAD54 translocases in eukaryotes, may be a conserved mechanism to limit off-target association and thereby favor recombinase binding to DSBR sites (90). Our surprising finding that the ability of *C. elegans* RAD-54.B to antagonize RAD-51 binding to unbroken DNA does not depend on its ATPase activity represents a departure from this scenario, suggesting that the mechanisms by which members of the RAD54 translocase family enhance binding fidelity of recombinases have diverged and expanded over evolution.

Furthermore, our study also yielded another new and unexpected plot twist, namely that RAD-54.L itself is largely responsible for promoting the ectopic hyperaccumulation of RAD-51 on unbroken DNA when RAD-54.B is absent during *C. elegans* meiosis. This unexpected finding that RAD-54.L promotes unproductive association of RAD-51 with unbroken DNA (in the *rad-54.B* null mutant background) represents an important *in vivo* demonstration that a RAD54.L ortholog can act on RAD51 in opposing ways, *i.e*. both to promote recombinase removal and to promote or stabilize recombinase association with DNA. While several studies have established that yeast Rad54 (RAD54.L ortholog) can stabilize Rad51 filaments *in vitro* (18,22), there has been less evidence regarding the *in vivo* occurrence and relevance of filament-stabilizing activity. Thus, our observation that RAD-54.L is required for RAD-51 hyperaccumulation on unbroken DNA in the *rad-54.B* mutant helps to address this gap, providing evidence for its capacity to stabilize RAD-51 filaments *in vivo*. Interestingly, our analysis of the ATPase-dead allele, *rad-54.L(K238R)*, indicated that such RAD-51 promoting/stabilizing activity of RAD-54.L is partially dependent on RAD-54.L ATPase activity. We speculate that this reflects both ATPase-independent RAD-51-stabilizing activity, as well as ATPase-dependent RAD-51-promoting activity, perhaps involving translocation along and opening of dsDNA (21). Further, our data imply that opposing RAD-54.L activities can operate even within the same nucleus, with RAD-54.L promoting RAD-51 removal and progression of recombination at *bona fide* DSBR sites while simultaneously facilitating association of RAD-51 with unbroken DNA elsewhere in the nucleus. Importantly, however, our data clearly indicate that RAD-54.L is *not* required to promote/stabilize RAD-51 association with DNA at meiotic DSBR sites, as evidenced by the abundant DSB-dependent accumulation and persistence of RAD-51 in *rad-54.L* mutants.

How might progression through the meiotic program influence or be served by the observed disparate activities of RAD-54.L and division of labor between RAD-54.L and RAD-54.B? Our thinking in this regard is informed by our findings that: (i) the unproductive hyperaccumulation of RAD-51 at unbroken DNA in the *rad-54.B* mutant occurs in parallel with productive association of RAD-51 at meiotic recombination intermediates and (ii) RAD-51 hyperaccumulation occurs specifically in the ‘CHK-2 active zone’ of the germ line and is dependent on CHK-2 activity. Several prior studies have shown that the window of activation of *C. elegans* CHK-2 from meiotic prophase onset through mid-pachytene corresponds to both the period of active DSB formation (49,68,76) as well as the timing of engagement of a specialized ‘meiotic mode’ of DSB repair involving modifications to the molecular requirements for processing and HR-mediated repair of DSBs (78,79). We hypothesize that RAD-54.L may become hyperactivated within the CHK-2 active window as part of the meiotic mode of DSBR. We speculate that enhanced activity of this key recombination factor contemporaneously with DSB formation may be beneficial for promoting efficient homology search and strand exchange to enable both crossover formation and restoration of genome integrity. However, hyperactivation of RAD-54.L to augment DSBR may also increase its ability to stabilize or promote the formation of unproductive RAD-51 filaments on unbroken DNA, thereby necessitating the proposed RAD-51 sequestering activity of RAD-54.B to antagonize this unproductive association.

The proposed hyperactivation of RAD-54.L during *C. elegans* meiosis stands in striking contrast to the previously-described regulation of the orthologous proteins in *S. cerevisiae* meiotic cells, which actively down-regulate the strand-exchange activity of Rad51, in part through Rad54-phosphorylation-mediated inhibition of Rad54-Rad51 complex formation (91). However, these opposing modes of regulation of the RAD51 recombinase/RAD54L translocase partnership make sense in light of notable differences in the inventory of meiotic machinery components present in these two organisms. In *S. cerevisiae*, downregulation of Rad51 recombinase activity during meiotic prophase is important to enable interhomolog strand-exchange driven by the meiotic recombinase Dmc1 (in partnership with Rdh54). In contrast, *C. elegans* lacks DMC1, so RAD-51 is the sole recombinase available to create the crossovers needed to segregate homologous chromosomes and to promote HR-mediated repair of meiotic DSBs.

Together, the findings presented here contribute to a growing appreciation of RAD54.L and RAD54.B as highly versatile components in the recombination toolbox. Depending on the context in which they are deployed and how they are regulated, the diverse biochemical activities demonstrated for these RAD54 paralogs can serve to augment, modulate and/or counteract the activities of recombinases, thereby protecting genome integrity by ensuring repair outcomes that are achievable and appropriate for the situation at hand.

## Supplementary Material

gkad638_Supplemental_File

## Data Availability

All 32-bit images, output from the foci quantification analysis, and custom Python script used for image analysis are available at the Biostudies Database (accession number S-BSST979, url: https://www.ebi.ac.uk/biostudies/studies/S-BSST979) (69). Materials used in this study are available upon request from AMV.
