# Supplementary material for: Disparate roles for C. elegans DNA translocase paralogs RAD-54.L and RAD-54.B in meiotic prophase germ cells
Source: Nucleic Acids Res. 2023 Aug 7;51(17):9183–202. doi: 10.1093/nar/gkad638 (PMC10516670; doi:10.1093/nar/gkad638)
Supplement: gkad638_Supplemental_File [file gkad638_supplemental_file.pdf]

Supplemental Information
Title: Disparate roles for *C. elegans* DNA translocase paralogs RAD-54.L and RAD-54.B in meiotic prophase germ cells

Authors: Kei Yamaya[1], Bin Wang[2], Nadin Memar[3], Arome Solomon Odiba[2], Alexander Woglar[1,4], Anton Gartner[3], Anne M. Villeneuve[1,5]

[1] Department of Developmental Biology, Stanford University School of Medicine, Stanford, California, USA
[2] State Key Laboratory of Non-food Biomass and Enzyme Technology, Guangxi Academy of Sciences, 530007 Nanning, China
[3] IBS Center for Genomic Integrity and Department for Biological Sciences, Ulsan National Institute of Science and Technology, Ulsan, Korea
[4] Swiss Institute for Experimental Cancer Research (ISREC), School of Life Sciences, Swiss Federal Institute of Technology Lausanne (EPFL), Lausanne, Switzerland (current affiliation)
[5] Department of Genetics, Stanford University School of Medicine, Stanford, California, USA

Corresponding author: Anne M. Villeneuve (annev@stanford.edu)

This PDF file includes Supplemental Text, SI references, Table S1, and Figures S1-S14.

Supplementary Information Text

Supplemental Materials and Methods

CRISPR-Cas9 Gene Editing

rad-54.B(gt3402[rad-54.B::GFP]) was generated by CRISPR-Cas9 gene editing using sgRNA(GAATGCCGGGTTGTCGCAGG). The PU6::sgRNA template was generated by PCR as described in (1). 5' and 3' homology arms were generated from N2 genomic DNA by PCR (primer sequences below) and then inserted into the GFP^SEC^3xFlag vector pDD282 using Gibson assembly (New England BioLabs). A mixture of pDD162 (Peft-3::Cas9 , 50 ng/µL), pCFJ90 (Pmyo-2 :: mCherry , 2.5 ng/µL) and pCFJ104 (Pmyo-3::mCherry , 5 ng/µL) plasmids (2), together with pU6-rad-54B sgRNA (50 ng/µL) and rad-54B:gfp repair templates (50 ng/µL), was microinjected in N2 young adult worms, followed by the screening, SEC excision and DNA sequencing as previous described in (3).
PCR primers for generation of 5' homology arm:
F: acgttgtaaaacgacggccagtcgccggcaCATCGATGCTCCTGAGGCTCCCGATGCTCC
R: CATCGATGCTCCTGAGGCTCCCGATGCTCCGgagcatcgggagcctcaggagcatcgatg
PCR primers for generation of 3' homology arm:
F: CGTGATTACAAGGATGACGATGACAAGAGATGATATTGATCTGTTGAATTTGTTT
R: ggaaacagctatgaccatgttatcgatttcCGCCGTAAATCTACCCTC

rad-54.L(me177) was generated using CRISPR-Cas9 gene editing as in (4). An alignment of *S. cerevisiae* Rad54 and *C. elegans* RAD-54.L was used to design the missense mutation in *rad-54.L(me177)*, which encodes RAD-54.L(K238R) with the identical amino acid substitution at the corresponding position present in the *S. cerevisiae* ATPase-dead mutant Rad54(K341R) (5, 6). sgRNA (ATGGCAGATGAAATGGGTCT) and ssDNA repair template (ATTAATATTCCCGAATTTCATGGATGTATTATGGCAGATGAAATGGGTCTTGGAAG GACACTTCAATGCATTTCTCTACTTTGGACACTTCT) (IDT) were used for microinjection into the germlines of WT animals. Animals carrying the edit were identified using PCR (F: GCGTCCGCATCAAAGAGATG, R: TCGAAACTGTTGGAC ACGCA) followed by a CviKI1 restriction enzyme digest. The edit was confirmed by Sanger sequencing.

rad-54.B(gt3328) was generated by CRISPR-Cas9 gene editing as described previously using Cas9 protein (0.25µg/µL), tracrRNA (0.02µg/µL, and crRNA (0.02µg/µL) (5'-AACCACGTCAGCAGTCACTG[TGG]-3'), *rol-6(su1006)* (40ng/µL) (7). A ssODN (0.11µg/µL) was used as the repair template with 40 bp homology arms flanking a 43bp universal STOP-in cassette (8) (5'GAACAGGATATTC TCGATCGAAAAACCACGTCAGCAGTCAGGGAAGTTTGTCCAGAGCAGAGGTGACT AAGTGATAAGCTAGCCTGTGGACACGAGATTCGCGAGACATCTCCGACCTCATCA G-3'). F1 rollers were screened by PCR using the following primers (5'-CGTCTGTAAACTCTTT CCGAG-3' and 5'-GGGTGGAGCCTAAATTTAAG-3'). Successful insertion resulted in a 513 bp fragment (WT fragment 470 bp) and was verified by sequencing.

rad-54.B(gt3379) was generated by CRISPR-Cas9 gene editing as described previously using Cas9 protein (0.25μg/μL), tracrRNA (0.02μg/μL, and crRNA (0.02μg/μL) (5'-CGGAAAGAGTTTACAGACGA[TGG]-3'), *rol-6(su1006)* (40ng/μL) (7). A ssODN (0.11μg/μL) was used as the repair template (5'-CCGGGAAAAACGGTGGTGGTGGCGGAGCA ATTCTCGCCGACGATATGGGACTCGGAGCTAGTTTACAGACCATGGCTGCCACGTGGGCTTTGTTGAAA GGTTAACCATTTTGTGG-3'). F1 rollers were screened by PCR (1412bp) and sequenced using the following primers (5'- GGGTGGAGCCTAAATTTAAG-3' and 5'-GTTGTTGGGCGGTTTTTGAGC-3'). Successful insertion was verified by PCR and sequencing over several generations to ensure homozygosity.

Meiotic crossover (CO) distribution assay using SNP mapping strategy
Meiotic CO distribution was assayed as described (9). TG3319(*rad-54.B(gk340656)*) was crossed to CB4856 to generate a new strain XSW933 homozygous for the *rad-54B(gk340656)* mutation as well as the CB4856-derived alleles at SNP sites, V-17.5, V-5, V5.8, V17.8, V25 (10) (**Table S1**). We then crossed TG3319 males to XSW933 young adult hermaphrodites, and crossed N2 males to CB4856 young adult hermaphrodites as a control. Following successful mating (~12 hours), L4 hermaphrodite worms of the F_1 generation from both crosses were individually picked and allowed to grow to the young adult stage. These hermaphrodites were mated to CB5584 males (carrying a pharyngeal GFP marker). After ~12 hours of mating, the F_1 animals were plated to lay eggs for two days. F1 animals were picked for single-worm lysis and genotyping by PCR. From the F_1 plates that were confirmed to be heterozygous for the CB4856-derived alleles of the chromosome *V* SNP markers , 256 F_2 cross-progeny L4 hermaphrodites (expressing the pharyngeal GFP marker) were picked. Single worm lysis was carried out and the V-17.5, V-5, V5.8, V17.8, V25 SNP sites were tested for presence of the CB4856-derived alleles by PCR. The primers used for the PCR are indicated in **Table S1**. The PCR reaction was set up at 94°C for 40 seconds, followed by 35 cycles of 40 seconds at 60°C and 1 minute at 72°C. This was followed by 10 minutes at 72°C. PCR products were digested with Takara Quickcut^{TM} Dra I (Aha III) restriction enzyme according to the manufacturer's instruction, then resolved on a 2% agarose gel (Sangon Biotech) for 40 minutes.

Supplemental References

1. Ward,J.D. (2015) Rapid and precise engineering of the *Caenorhabditis elegans* genome with lethal mutation co-conversion and inactivation of NHEJ repair. *Genetics*, **199**, 363–377.

2. Dickinson,D.J., Ward,J.D., Reiner,D.J. and Goldstein,B. (2013) Engineering the *Caenorhabditis elegans* genome using Cas9-triggered homologous recombination. *Nat. Methods*, **10**, 1028–1034.

3. Dickinson,D.J., Pani,A.M., Heppert,J.K., Higgins,C.D. and Goldstein,B. (2015) Streamlined genome engineering with a self-excising drug selection cassette. *Genetics*, **200**, 1035–1049.

4. Paix,A., Folkmann,A., Rasoloson,D. and Seydoux,G. (2015) High efficiency, homology-directed genome editing in *Caenorhabditis elegans* using CRISPR-Cas9 ribonucleoprotein complexes. *Genetics*, **201**, 47–54.

5. Clever,B., Schmuckli-Maurer,J., Sigrist,M., Glassner,B.J. and Heyer,W.D. (1999) Specific negative effects resulting from elevated levels of the recombinational repair protein Rad54p in *Saccharomyces cerevisiae. Yeast Chichester Engl.*, **15**, 721–740.

6. Petukhova,G., Komen,S.V., Vergano,S., Klein,H. and Sung,P. (1999) Yeast Rad54 promotes Rad51-dependent homologous DNA pairing via ATP hydrolysis-driven change in DNA double helix conformation *. *J. Biol. Chem.*, **274**, 29453–29462.

7. Dokshin,G.A., Ghanta,K.S., Piscopo,K.M. and Mello,C.C. (2018) Robust genome editing with short single-stranded and long, partially single-stranded DNA donors in *Caenorhabditis elegans*. *Genetics*, **210**, 781–787.

8. Wang,H., Park,H., Liu,J. and Sternberg,P.W. (2018) An efficient genome editing strategy to generate putative null mutants in *Caenorhabditis elegans* using CRISPR/Cas9. *G3 GenesGenomesGenetics*, **8**, 3607–3616.

9. Agostinho,A., Meier,B., Sonneville,R., Jagut,M., Woglar,A., Blow,J., Jantsch,V. and Gartner,A. (2013) Combinatorial regulation of meiotic Holliday Junction resolution in *C. elegans* by HIM-6 (BLM) helicase, SLX-4, and the SLX-1, MUS-81 and XPF-1 nucleases. *PLOS Genet.*, **9**, e1003591.

10. Davis,M.W., Hammarlund,M., Harrach,T., Hullett,P., Olsen,S. and Jorgensen,E.M. (2005) Rapid single nucleotide polymophism mapping in *C. elegans. BMC Genomics*, **6**, 1–11.

11. Chi,P., Kwon,Y., Seong,C., Epshtein,A., Lam,I., Sung,P. and Klein,H.L. (2006) Yeast recombination factor Rdh54 functionally interacts with the Rad51 recombinase and catalyzes Rad51 removal from DNA. *J. Biol. Chem.*, **281**, 26268–26279.

12. Rockman,M.V. and Kruglyak,L. (2009) Recombinational landscape and population genomics of *Caenorhabditis elegans*. *PLOS Genet.*, **5**, e1000419.

<u>Table S1</u>: Dra I SNP locations and primers sequences for CO distribution assay.

Location	primers
V -17.5 F	GCGACTGTCACAATCAAGA
V -17.5 R	CTGCTTGGCTTCCTCTA
V -5 F	GAGATTCTAGAGAAATGGACACCC
V -5 R	AAAAATCGACTACACCACTTTTAGC
V 5.8 F	CCTTATCTAGTAATTTGCCTGTTGT
V 5.8 R	ACATAAGCGCCATAACAAGTCG
V 17.8 F	GAAATTCAAATTTTTGAGAAACCC
V 17.8 R	TTCAGACCATTTTTAGAATATTCAGG
V 25.1 F	ACTTGACTCCTCTTTTCCATG
V 25.1 R	CTGCTAGCTCAAATACTCCC

Fig S1

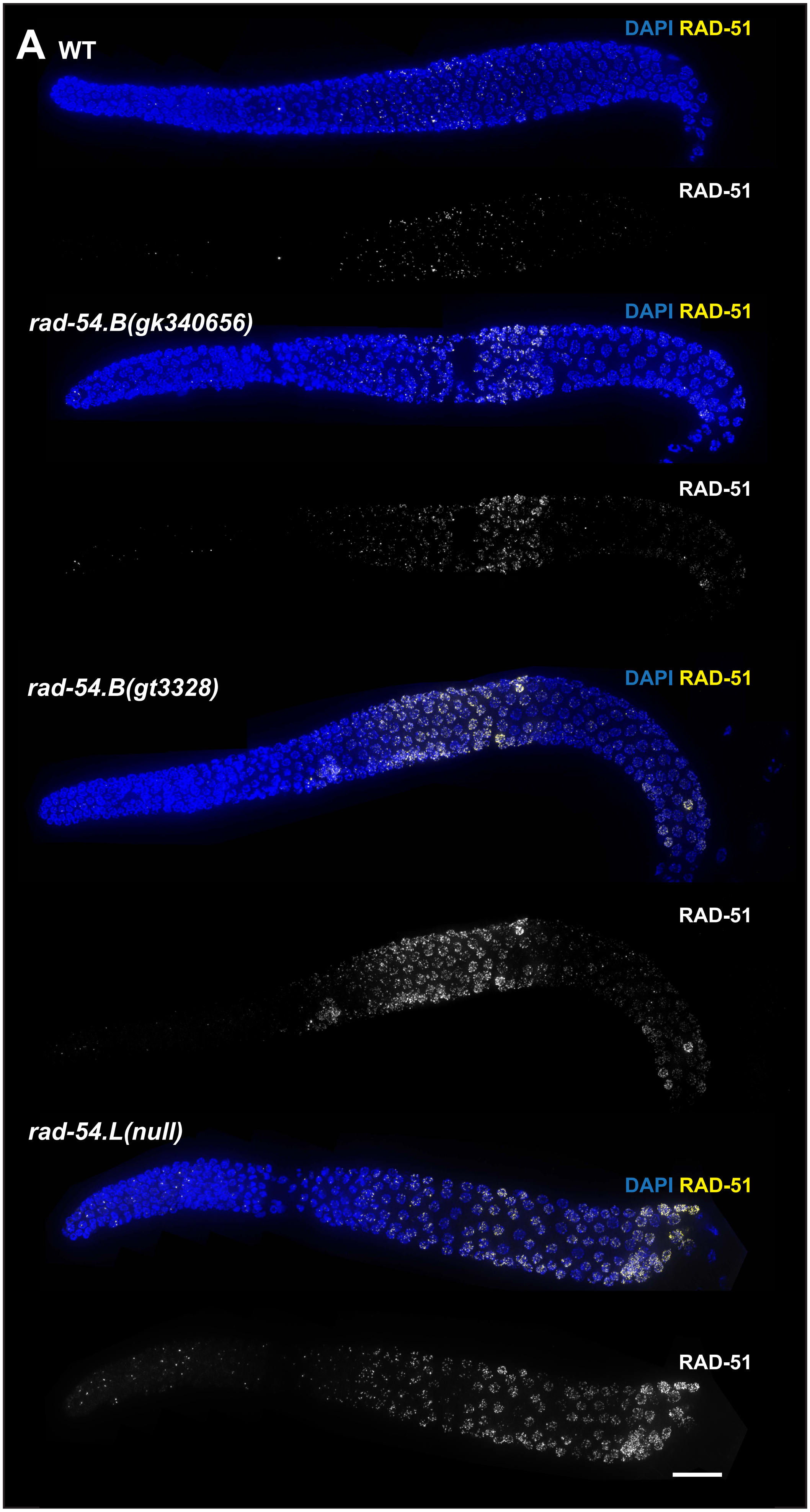

Fig S1. **Hyperaccumulation of RAD-51 in *rad-54.B* mutants differs from the *rad-54.L* mutant.** A) Max-projected images of whole-mount gonads from indicated genotypes immunostained for RAD-51. Full genotype of *rad-54.L(null)*: *rad-54.L(me98)*. While both *rad-54.B* and *rad-54.L* mutants have elevated levels of RAD-51 foci, in both *rad-54.B* mutants, elevated RAD-51 foci decline midway through meiotic progression, whereas RAD-51 foci continue to accumulate in the *rad-54.L* mutant. Scale bar represents 20 μm.

Fig S2

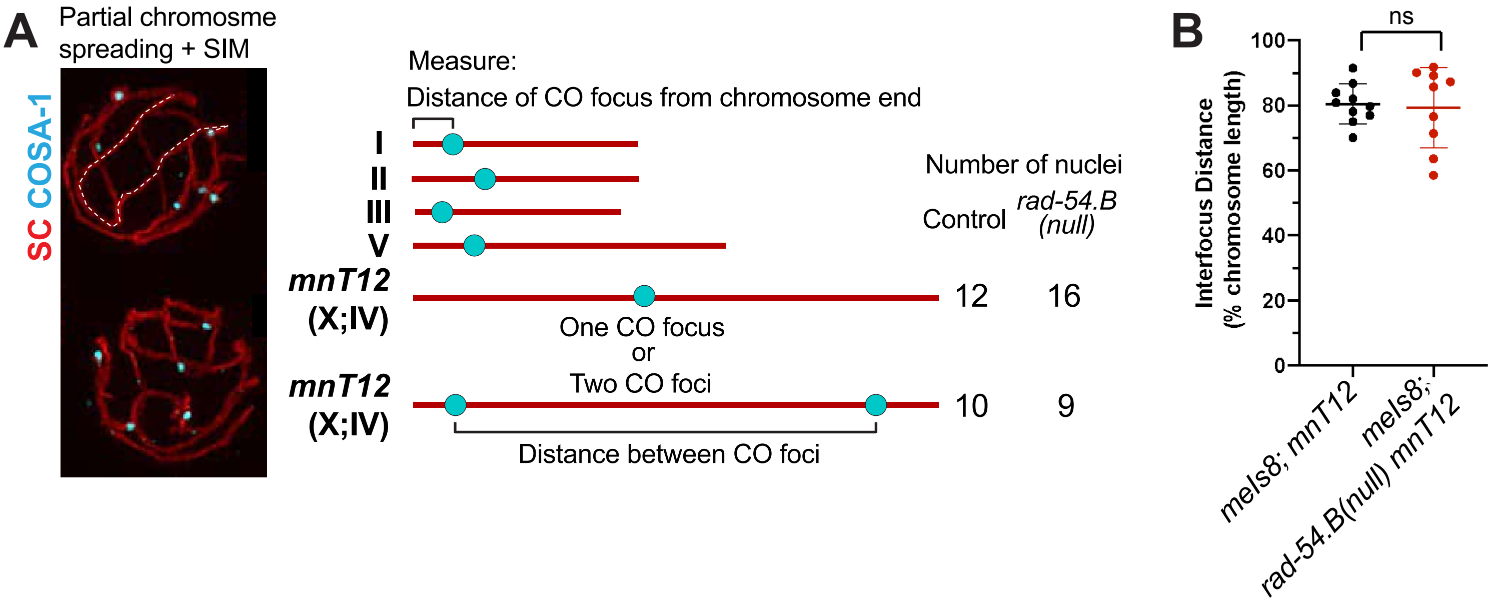

Fig S2. **Normal spacing between CO-site foci in the rad-54.B mutant background.** A) Left: Examples of late pachytene nuclear spreads, imaged with SIM, that were used for chromosome tracing and quantification of positions of CO-designated recombination sites marked by immunolocalization of GFP::COSA-1 (cyan) and MSH-5 (not depicted). Middle: Schematic of the karyotype of the strains analyzed (control: *meIs8[pie-1p::GFP::cosa-1::unc-119(+)]; mnT12*, and *rad-54.B*: *meIs8; rad-54.B(gk340656) mnT12*) and the features quantified. Right: Numbers of nuclei assayed in control (*meIs8; mnT12*) and *rad-54.B* (*meIs8; rad-54.B mnT12*) backgrounds that had one or two CO foci on the *mnT12* fusion chromosome bivalent. B) Quantification of inter-focus distances on *mnT12* fusion chromosome bivalents that had 2 CO site foci, represented as a percentage of the total *mnT12* chromosome length. Control, n=10 chromosomes; *rad-54.B*, n=9 chromosomes. Statistical significance was assessed with a Mann Whitney test; ns, $p>0.05$.

Fig S3

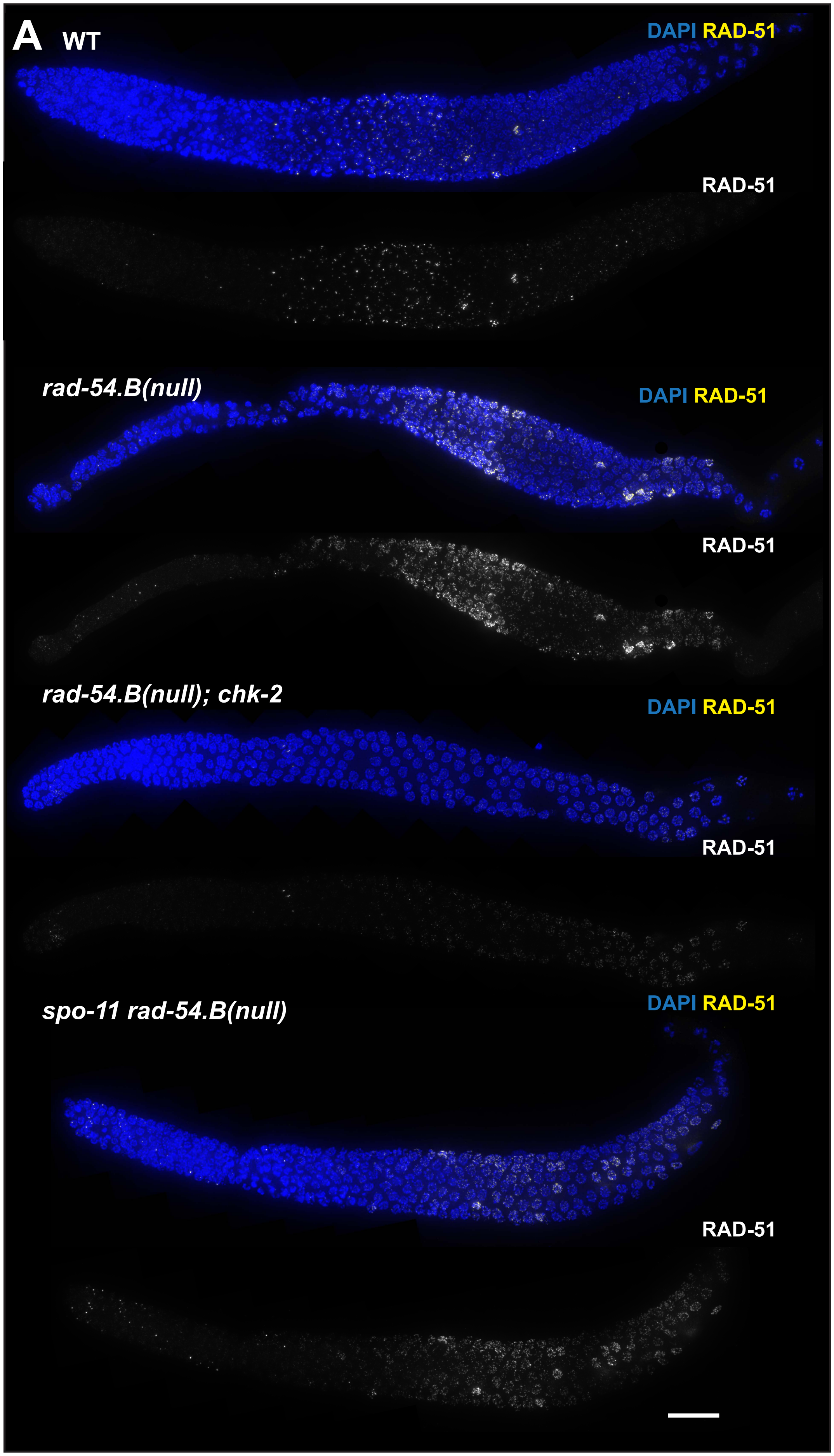

Fig S3. **Hyperaccumulation of RAD-51 foci in *rad-54.B* background is dependent on CHK-2.** A) Max-projected images of whole-mount gonads from indicated genotypes immunostained for RAD-51. Full genotypes: WT, *rad-54.B(gk340656)(null); egl-1 yIs34 oxTi633*, *rad-54.B(null); egl-1 chk-2*, *spo-11 rad-54.B(null)*. We note that while there are some faint RAD-51 foci detected in the *rad-54.B; chk-2* mutant, these are far weaker, fewer in number and occur much later in prophase than the RAD-51 foci seen in WT, *rad-54.B*, or *spo-11 rad-54.B* germ lines, supporting the conclusion that the RAD-51 hyperaccumulation phenotype observed in *rad-54.B* mutants is dependent on CHK-2. Scale bar represents 20 μm.

Fig S4

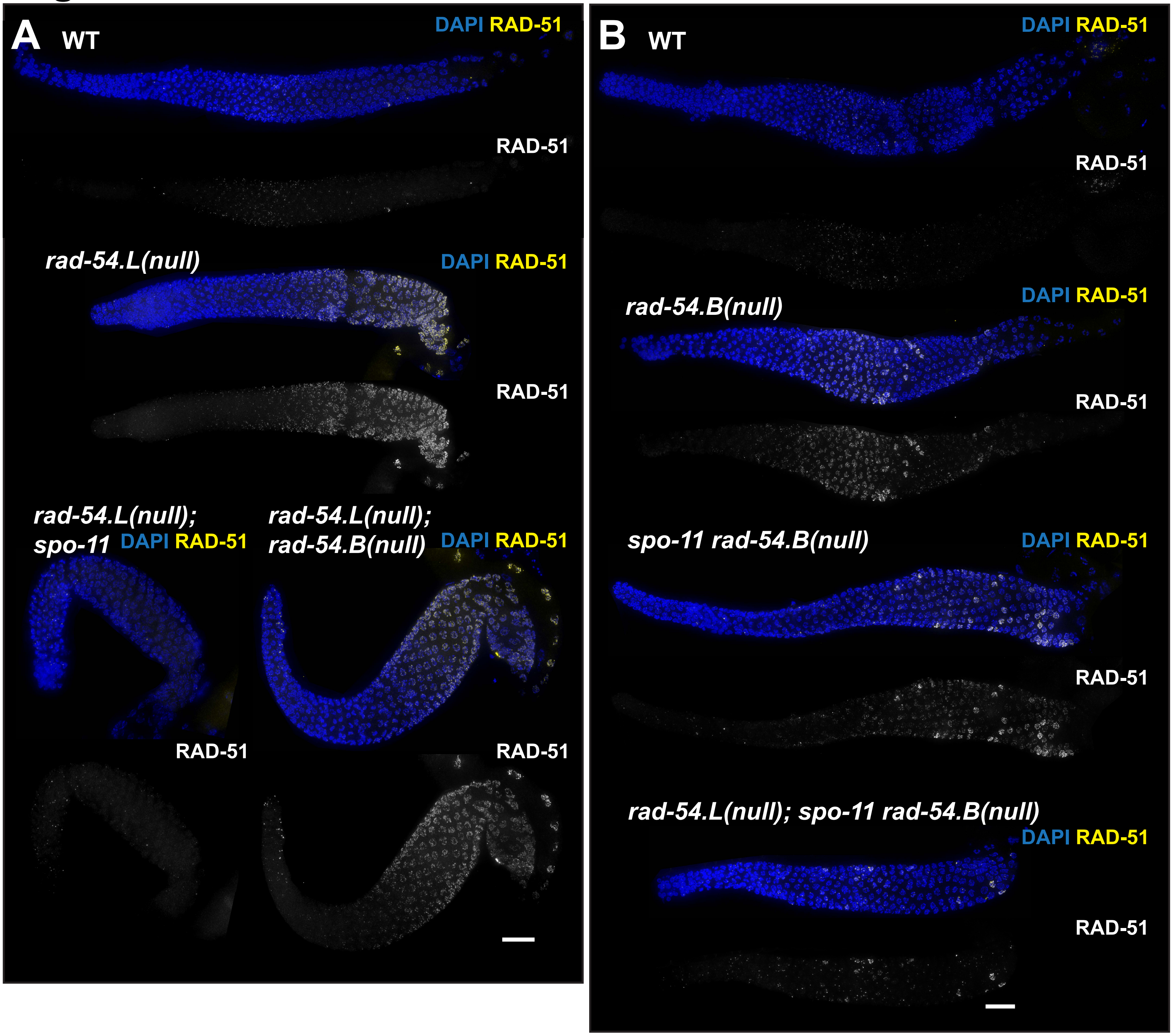

Fig S4. **RAD-51 hyperaccumulates at unbroken DNA in the *rad-54.B* mutant.** A) and B) Max-projected images of whole-mount gonads from indicated genotypes immunostained for RAD-51. Full genotypes: A), WT, *rad-54.L(me98)(null)*, *rad-54.L(null); spo-11(me44)*, *rad-54.L; rad-54.B(gk340656)(null)*. B), WT, *rad-54.B(gk340656)(null)*, *spo-11 rad-54.B(null)*, *rad-54.L(me98)(null), spo-11 rad-54.B(null)*. Scale bar represents 20 μm.

Fig S5

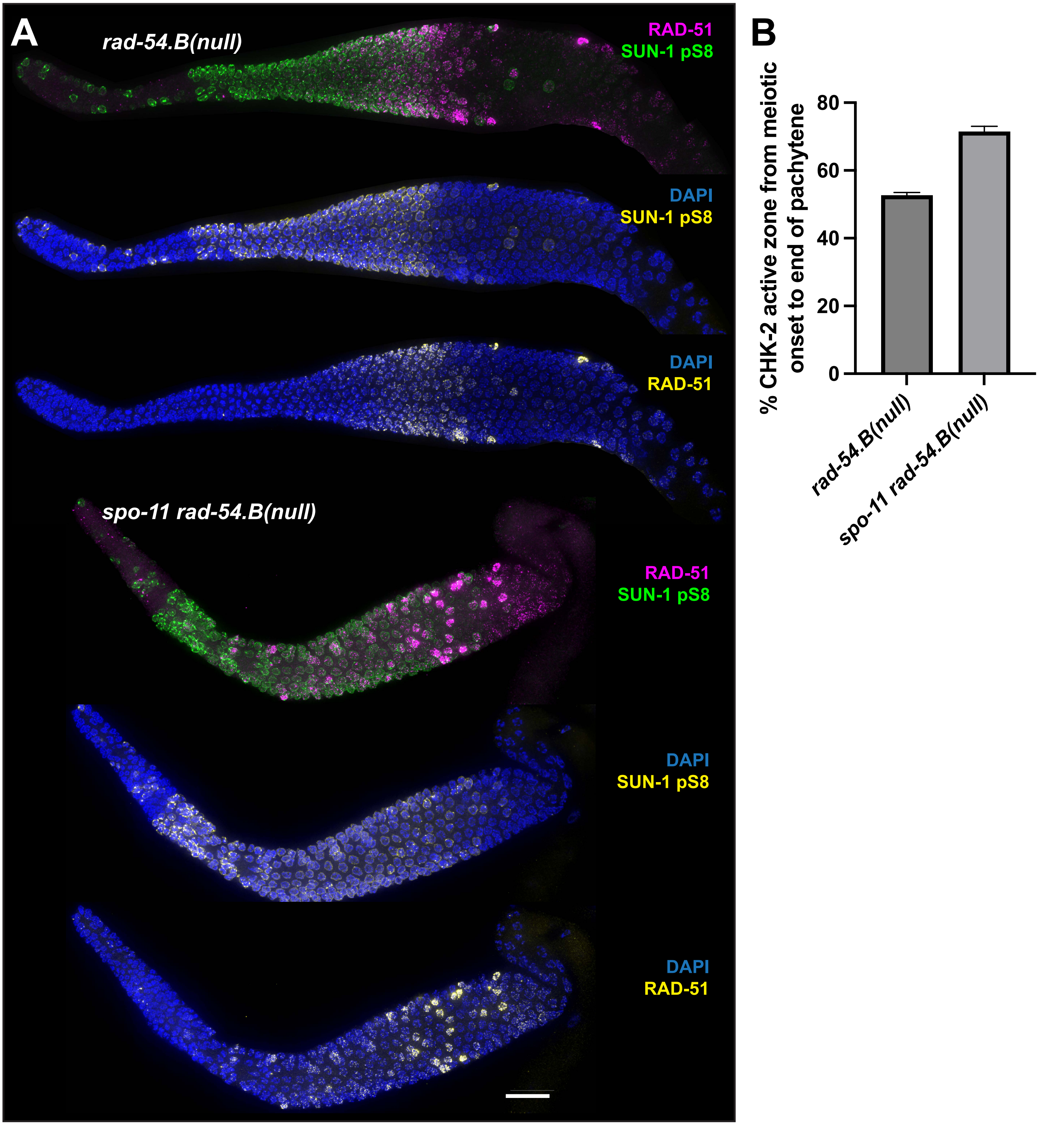

Fig S5. **RAD-51 hyperaccumulation zone is extended in *spo-11 rad-54.B* germlines relative to *rad-54.B* germlines.** A) Max-projected images of whole-mount gonads from indicated genotypes, immunostained for RAD-51 and SUN-1 pS8. Full genotypes: *rad-54.B(gk340656)(null)*, *spo-11(me44) rad-54.B(null)*. The zone of RAD-51 hyperaccumulation is extended in *spo-11 rad-54.B(null)* compared to *rad-54.B(null)*. This is partially attributable to extension of the CHK-2 active zone in the *spo-11 rad-54.B(null)* mutant, which reflects triggering of the "crossover assurance" checkpoint in the *spo-11* mutant background, and has been documented previously in several studies. We note that RAD-51 hyperaccumulation is extended for a few cell rows beyond the end of the CHK-2 active zone in the *spo-11 rad-54.B(null)* mutant for reasons unknown. Scale bar represents 20 µm. (B) Quantification of the CHK-2 active zone, defined as the contiguous region where the majority of nuclei in each cell row exhibits SUN-1 pS8 staining. Error bars represent standard deviation. Number of gonads analyzed: *rad-54.B(null)*, n=3; *spo-11 rad-54.B(null)*, n=3; unpaired t-test $p<0.0001$.

Fig S6

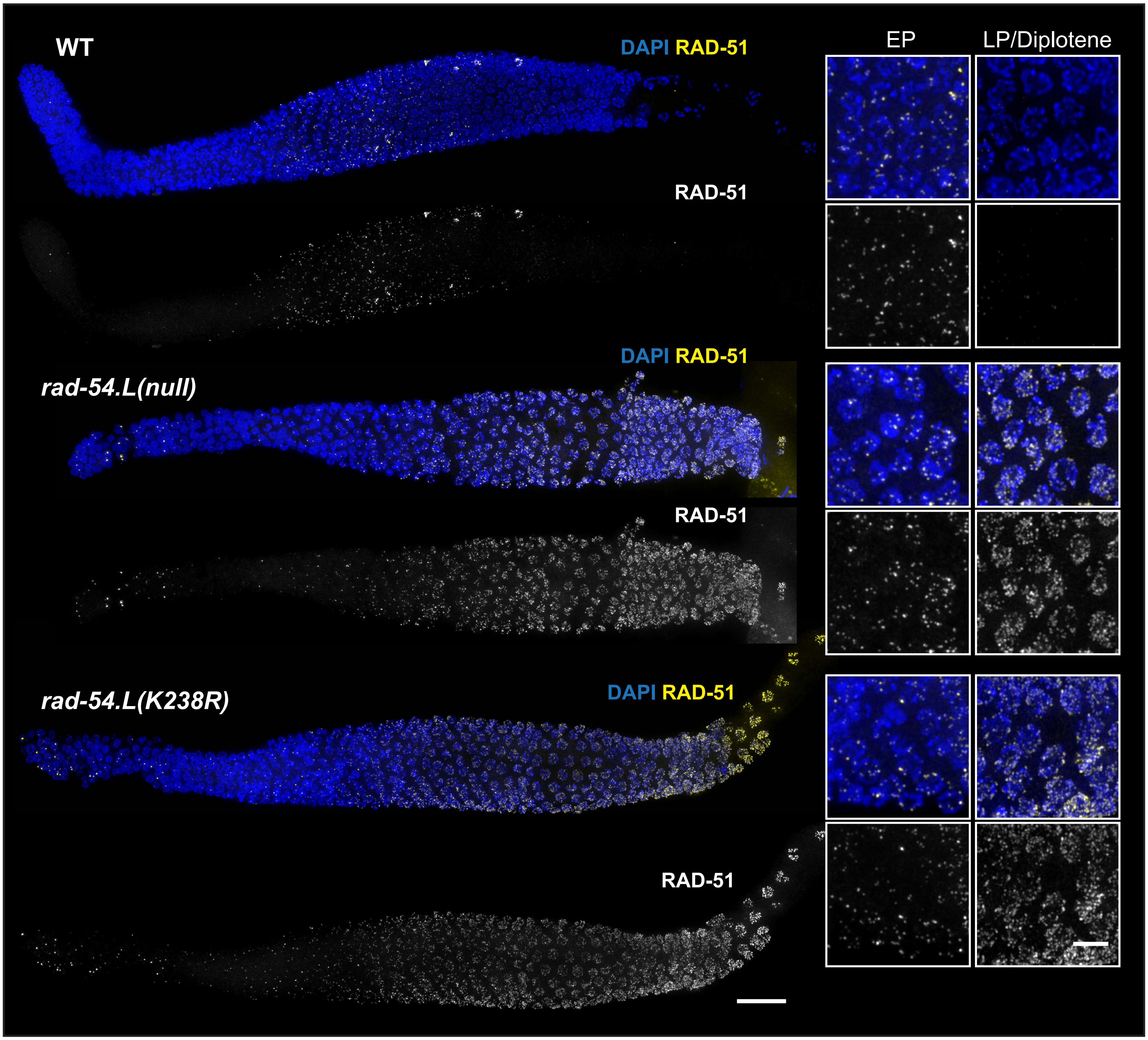

Fig S6. **RAD-51 hyperaccumulates in *rad-54.L(K238R)* germ lines similarly to *rad-54.L(me98)(null)* germ lines.** Max-projected images of whole-mount gonads from indicated genotypes immunostained for RAD-51. Full genotypes: WT, *rad-54.L(me98)(null)*, *rad-54.L(me177[K238R])*. Scale bar represents 20 µm. Insets at right depict zoomed-in fields of nuclei from early pachytene (EP) and late pachytene (LP)/diplotene stages. Scale bar in insets, 5 µm.

Fig S7

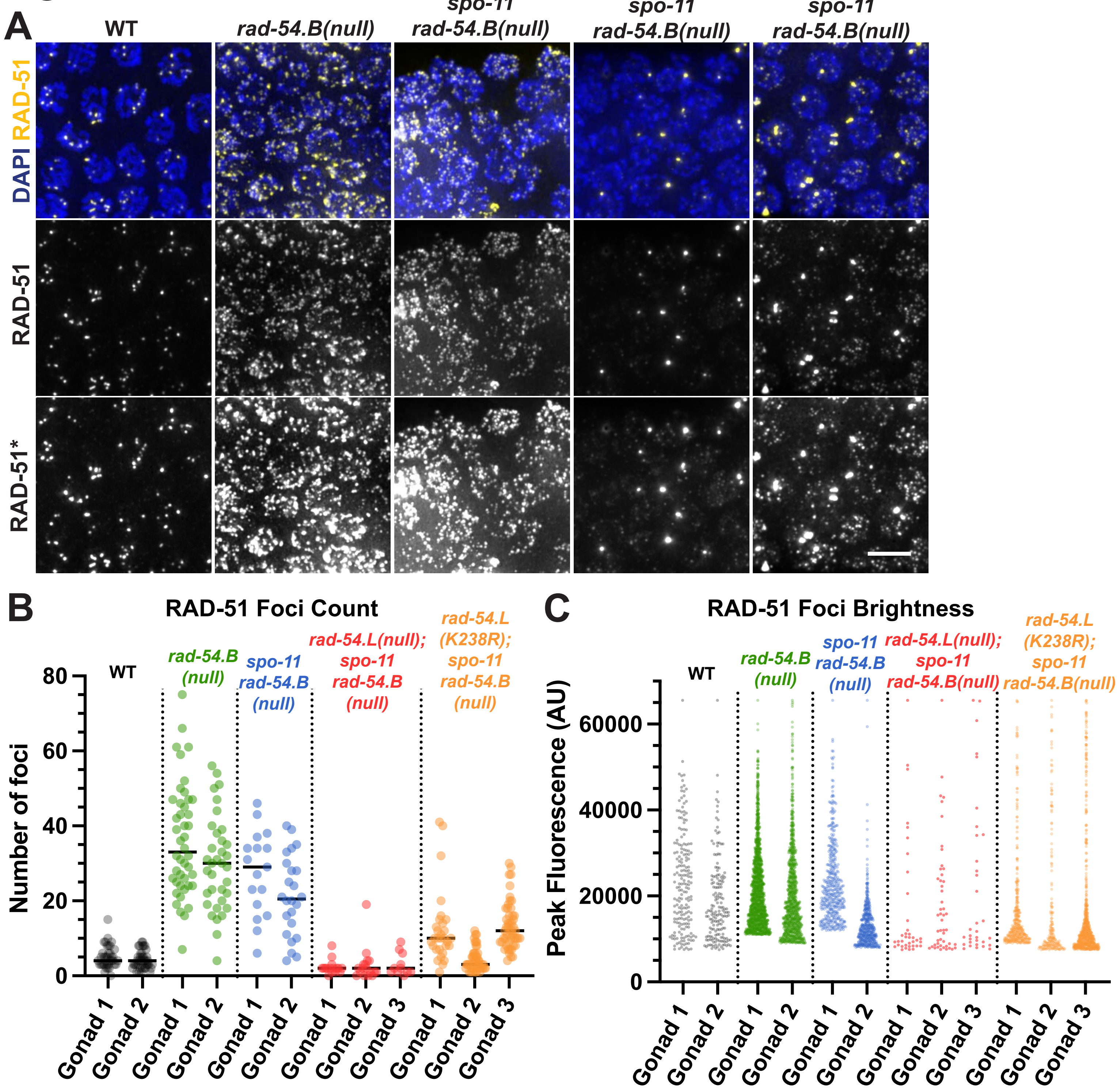

A
WT
rad-54.B(null)
spo-11 rad-54.B(null)
rad-54.L(null); spo-11 rad-54.B(null)
rad-54.L(K238R); spo-11 rad-54.B(null)
DAPI RAD-51
RAD-51
RAD-51*
B
RAD-51 Foci Count
Number of foci
0
20
40
60
80
WT
rad-54.B (null)
spo-11 rad-54.B (null)
rad-54.L(null); spo-11 rad-54.B (null)
rad-54.L (K238R); spo-11 rad-54.B (null)
Gonad 1
Gonad 2
Gonad 1
Gonad 2
Gonad 1
Gonad 2
Gonad 1
Gonad 2
Gonad 3
Gonad 1
Gonad 2
Gonad 3
C
RAD-51 Foci Brightness
Peak Fluorescence (AU)
0
20000
40000
60000
WT
rad-54.B (null)
spo-11 rad-54.B (null)
rad-54.L(null); spo-11 rad-54.B(null)
rad-54.L (K238R); spo-11 rad-54.B(null)
Gonad 1
Gonad 2
Gonad 1
Gonad 2
Gonad 1
Gonad 2
Gonad 1
Gonad 2
Gonad 3
Gonad 1
Gonad 2
Gonad 3

Fig S7**. Effect of *rad-54.L(K238R)* ATPase-dead mutant on SPO-11-independent accumulation of RAD-51.** A) Representative fields of nuclei from max-projected images of whole-mount gonads, taken from the zones of maximum accumulation of RAD-51 foci for the indicated genotypes; scale bar represents 5 μm. Comparison of images reveals: 1) a substantial attenuation of SPO-11-independent RAD-51 hyperaccumulation in *rad-54.L(K238R); spo-11(me44) rad-54.B(gk340656)(null)* relative to *spo-11 rad-54.B(null)*, and 2) more abundant residual SPO-11-independent RAD-51 foci in *rad-54.L(K238R); spo-11 rad-54.B(null)* relative to *rad-54.L(null); spo-11 rad-54.B(null)*. Bottom row depicts overexposure of RAD-51 signal (RAD-51*), which reveals very faint nuclear foci in *rad-54.L(null); spo-11 rad-54.B(null)* germ cells that are dimmer than those detected in *rad-54.L(K238R); spo-11 rad-54.B(null)* and are below the threshold intensity required for reliable quantification. B) Quantification of RAD-51 foci for the genotypes shown in panel A. RAD-51 foci counts were conducted in the zone of maximum accumulation of RAD-51 foci for each genotype. Each circle represents a nucleus; lines indicate median values. Numbers of nuclei analyzed (n) and median numbers of foci (m) were as follows: WT gonad 1, n =37, m=4, gonad 2, n=40, m=4; *rad-54.B(null)* gonad 1, n=44, m=33, gonad 2, n=35, m=30; *spo-11 rad-54.B(null)* gonad 1, n=17, m=29, gonad 2, n=24, m=20.5; *rad-54.L(null); spo-11 rad-54.B(null)* gonad 1, n=16, m=2, gonad 2, n=17, m=2, gonad 3, n=11, m=2; *rad-54.L(K238R); spo-11 rad-54.B(null)* gonad 1, n=30, m=10, gonad 2, n=45, m=3, gonad 3, n=50, m=12. C) Peak intensity of RAD-51 foci detected in the zone of maximum accumulation for each genotype. Each point in the scatter plot represents a RAD-51 focus. Note that quantification pipeline was run in some gonads with a higher threshold peak value to minimize false positive peaks (e.g. peaks identified in off-focus z-planes or in background control areas). Numbers of foci analyzed (n) were as follows: WT gonad 1, n=186, gonad 2, n=169; *rad-54.B(null)* gonad 1, n=1587, gonad 2, n=1047; *spo-11 rad-54.B(null)* gonad 1, n=467, gonad 2, n=509; *rad-54.L(null); spo-11 rad-54.B(null)* gonad 1, n=37, gonad 2, n=49, gonad 3, n=33; *rad-54.L(K238R); spo-11 rad-54.B(null)* gonad 1, n=370, gonad 2, n=201, gonad 3, n=669.

Fig S8

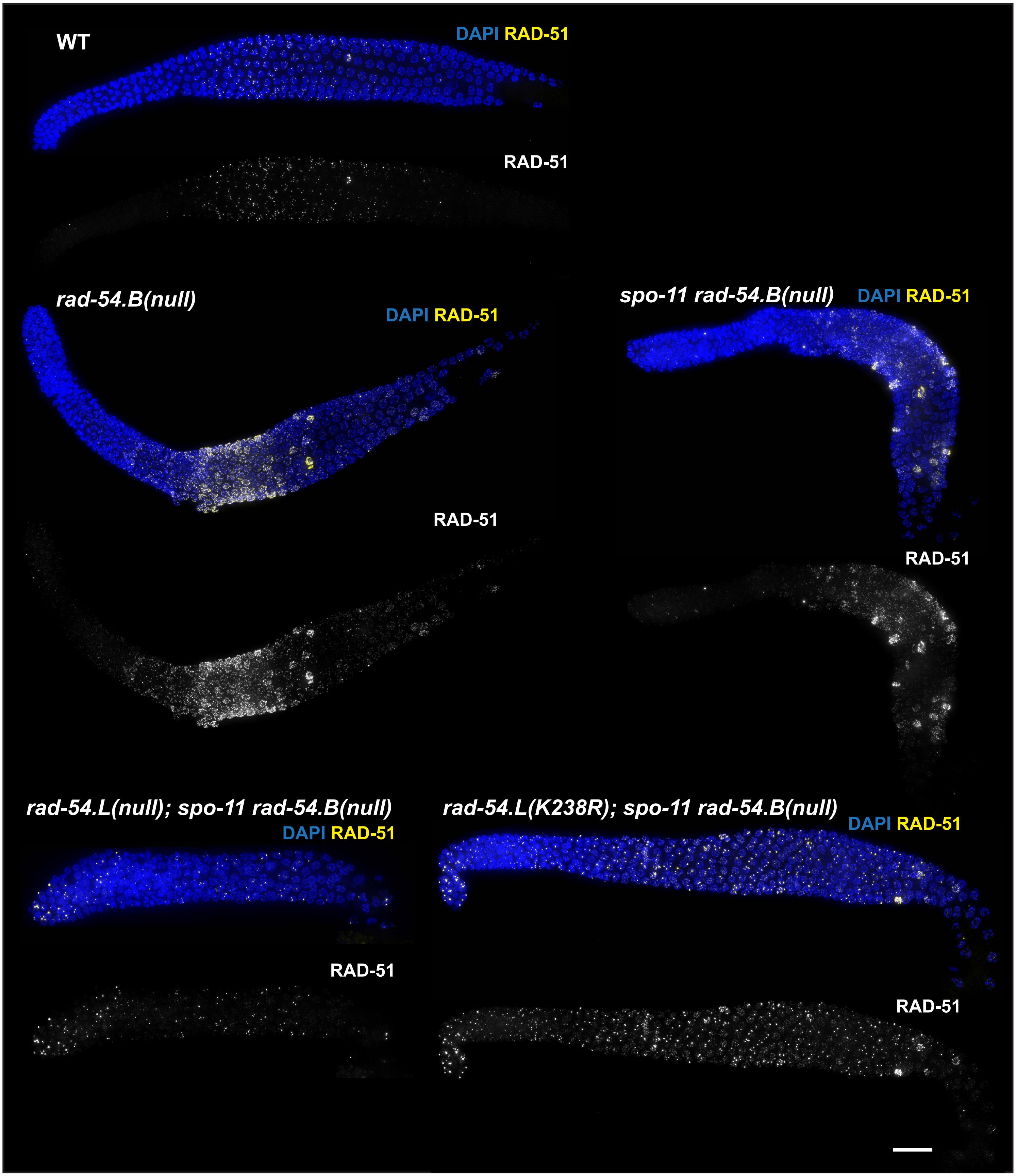

Fig S8. **Effect of *rad-54.L(K238R)* ATPase-dead mutant on SPO-11-independent accumulation of RAD-51 (continued)**. Max-projected images of whole-mount gonads from indicated genotypes immunostained for RAD-51. Full genotypes: WT, *rad-54.B(gk340656)(null)*, *spo-11(me44) rad-54.B(null)*, *rad-54.L(me98)(null); spo-11 rad-54.B(null)*, *rad-54.L(me177[K238R]); spo-11 rad-54.B(null)*. Scale bar represents 20 µm.

Fig S9

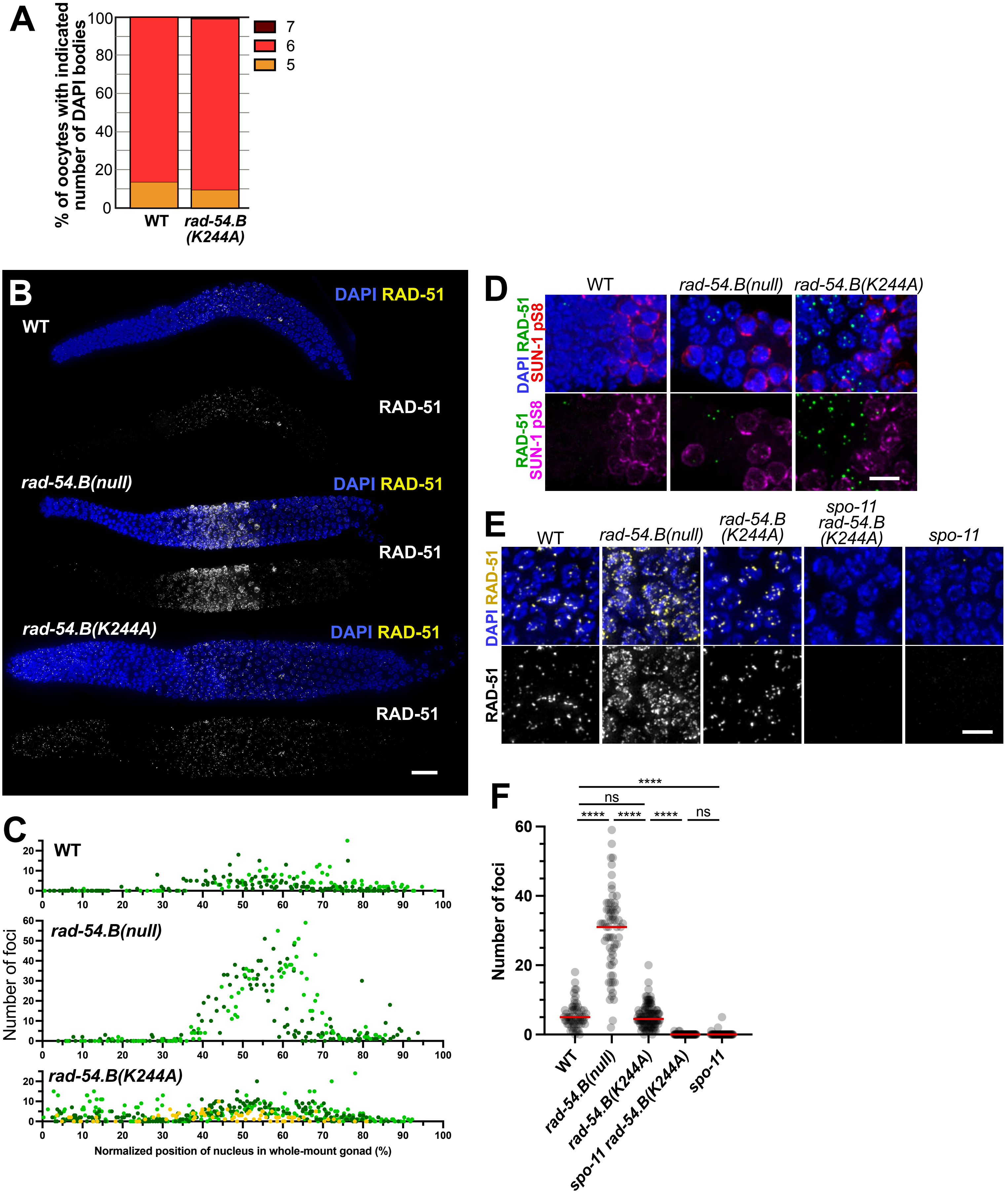
A
% of oocytes with indicated number of DAPI bodies
100
80
60
40
20
0
7
6
5
WT
rad-54.B (K244A)
B
WT
DAPI RAD-51
RAD-51
rad-54.B(null)
DAPI RAD-51
RAD-51
rad-54.B(K244A)
DAPI RAD-51
RAD-51
C
WT
rad-54.B(null)
rad-54.B(K244A)
Number of foci
Normalized position of nucleus in whole-mount gonad (%)
D
WT
rad-54.B(null)
rad-54.B(K244A)
DAPI RAD-51 SUN-1 pS8
RAD-51 SUN-1 pS8
E
WT
rad-54.B(null)
rad-54.B (K244A)
spo-11 rad-54.B (K244A)
spo-11
DAPI RAD-51
RAD-51
F
****
ns
****
****
****
ns
Number of foci
60
40
20
0
WT
rad-54.B(null)
rad-54.B(K244A)
spo-11 rad-54.B(K244A)
spo-11

Fig S9. **Analysis of ATPase-dead *rad-54.B(K244A)* mutant.** A) Quantification of diakinesis oocyte karyotypes in indicated genotypes. Number of oocytes assessed: WT, n=125; *rad-54.B(gt3379[K244A])*, n=126. Statistical significance assessed by Mann-Whitney test; WT vs *rad-54.B(K244A)*, ns (p>0.05). B) Representative max-projected images of WT, *rad-54.B(gk340656)(null)*, and *rad-54.B(K244A)* whole-mount gonads immunostained for RAD-51. Scale bar represents 20 μm. C) Quantification of RAD-51 foci for genotypes depicted in (B). Each data point represents an individual nucleus, with relative position calculated as the percentage of the length between the distal tip (0%) and the end of pachytene zone (100%); different colors represent the different gonads used for quantification. D) Max-projected images of germ cell nuclei from the region of the gonad spanning entry into meiotic prophase. SUN-1 pS8 signal at the nuclear envelope (primarily seen in nuclei on the right side of each image) reflects activation of CHK-2 and marks entry into meiotic prophase, whereas nuclei lacking SUN-1 pS8 signal have not yet entered meiotic prophase. Although meiotic RAD-51 foci were not elevated in the *rad-54.B(K244A)* mutant (see also E, F below), B-D illustrate variable detection of elevated *premeiotic* RAD-51 foci in this mutant. Observation of elevated (premeiotic) RAD-51 foci in the *rad-54.B(K244A)* mutant relative to the *rad-54.B(null)* mutant is reminiscent of a previously-published report showing that an ATPase-dead allele of yeast *rdh54(K352R)* causes a stronger DSBR defect than an *rdh54(null)* mutation (11); both observations suggest that the presence of a catalytically-inactive version of a RAD54B ortholog can in some contexts represent a greater impediment to DSBR than when the protein is absent. Scale bar represents 5 μm. E) Max-projected images of early pachytene nuclei stained for RAD-51 for indicated genotypes. Scale bar represents 5 μm. F) Quantification of early pachytene RAD-51 foci for immunostained germ cells represented in (E). WT, n=60, m=5; *rad-54.B(null)*, n=65, m=31; *rad-54.B(K244A)*, n=110, m=4.5; *spo-11(me44) rad-54.B(K244A)*, n=73, m=0; *spo-11*, n=57, m=0. Statistical significance was assessed with a Mann Whitney test; ns, p>0.05; ****, p<0.0001.

Fig S10

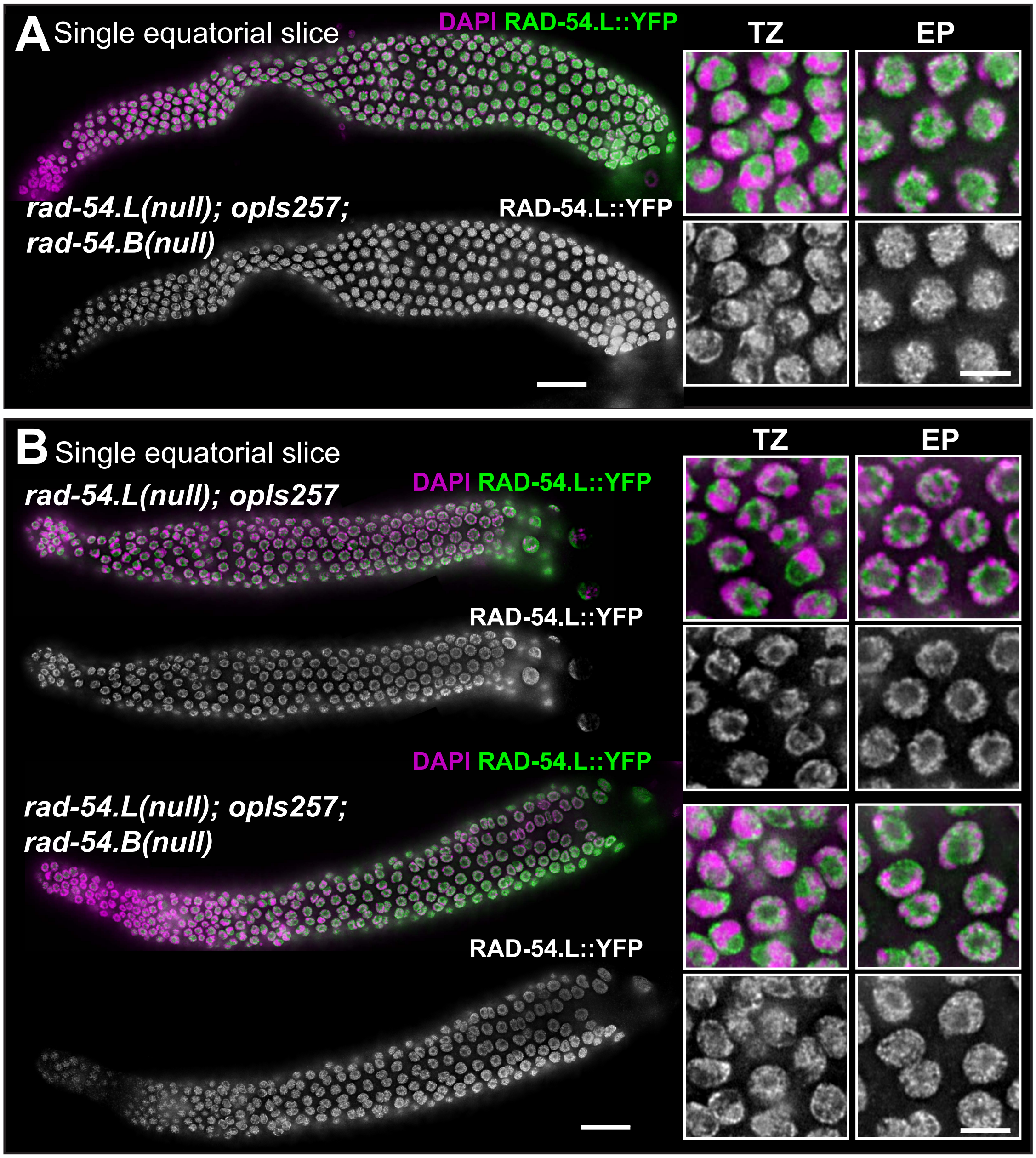

Fig S10. **RAD-54.L::YFP localization.** A) Image of whole-mount gonad from a *rad-54.L(me98)(null); opIs257[rad-54.Lp::rad-54.L::YFP::rad-54.L 3'UTR + unc-119(+)]; rad-54.B(null)* worm immunostained for RAD-54.L::YFP (using GFP antibody). The image represents a single z-slice showing an equatorial view of nuclei (instead of a max-projection). Scale bar represents 20 µm. Insets at right depict zoomed-in fields of nuclei from the transition zone (TZ) and early pachytene zone (EP). Scale bar in insets, 5 µm. B) Image of whole-mount gonad immunostained for RAD-54.L::YFP from worms of the indicated genotypes. Full genotypes are as detailed in Fig. 5A and S8A. The image represents a single z-slice showing an equatorial view of nuclei (instead of a max-projection). Unlike in Fig. 5A and S8A, these images show examples of gonads in which RAD-54.L::YFP signal is not observed in the nucleolus. Scale bar represents 20 µm. Insets at right depict zoomed-in fields of nuclei from the transition zone (TZ) and early pachytene zone (EP). Scale bar in insets, 5 µm.

Fig S11

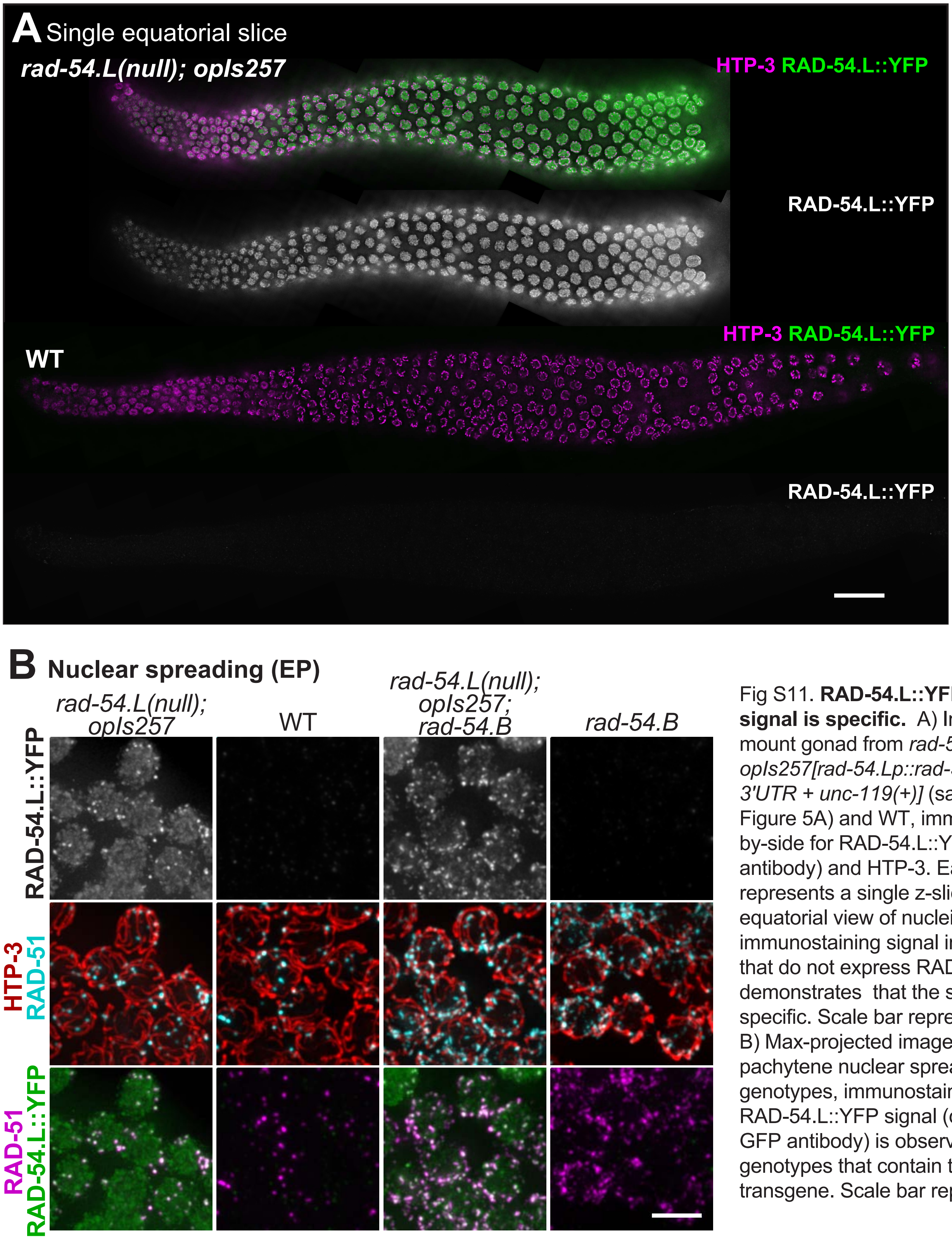

Fig S11. **RAD-54.L::YFP immunostaining signal is specific.** A) Image of whole-mount gonad from *rad-54.L(me98)(null); opIs257[rad-54.Lp::rad-54.L::YFP::rad-54.L 3'UTR + unc-119(+)]* (same sample as in Figure 5A) and WT, immunostained side-by-side for RAD-54.L::YFP (using GFP antibody) and HTP-3. Each image represents a single z-slice showing an equatorial view of nuclei. Lack of GFP immunostaining signal in control gonads that do not express RAD-54.L::YFP demonstrates that the signal observed is specific. Scale bar represents 20 μm. B) Max-projected images of early pachytene nuclear spreads of indicated genotypes, immunostained side-by-side. RAD-54.L::YFP signal (detected using GFP antibody) is observed only in the genotypes that contain the *opIs257* transgene. Scale bar represents 5 μm.

Fig S12

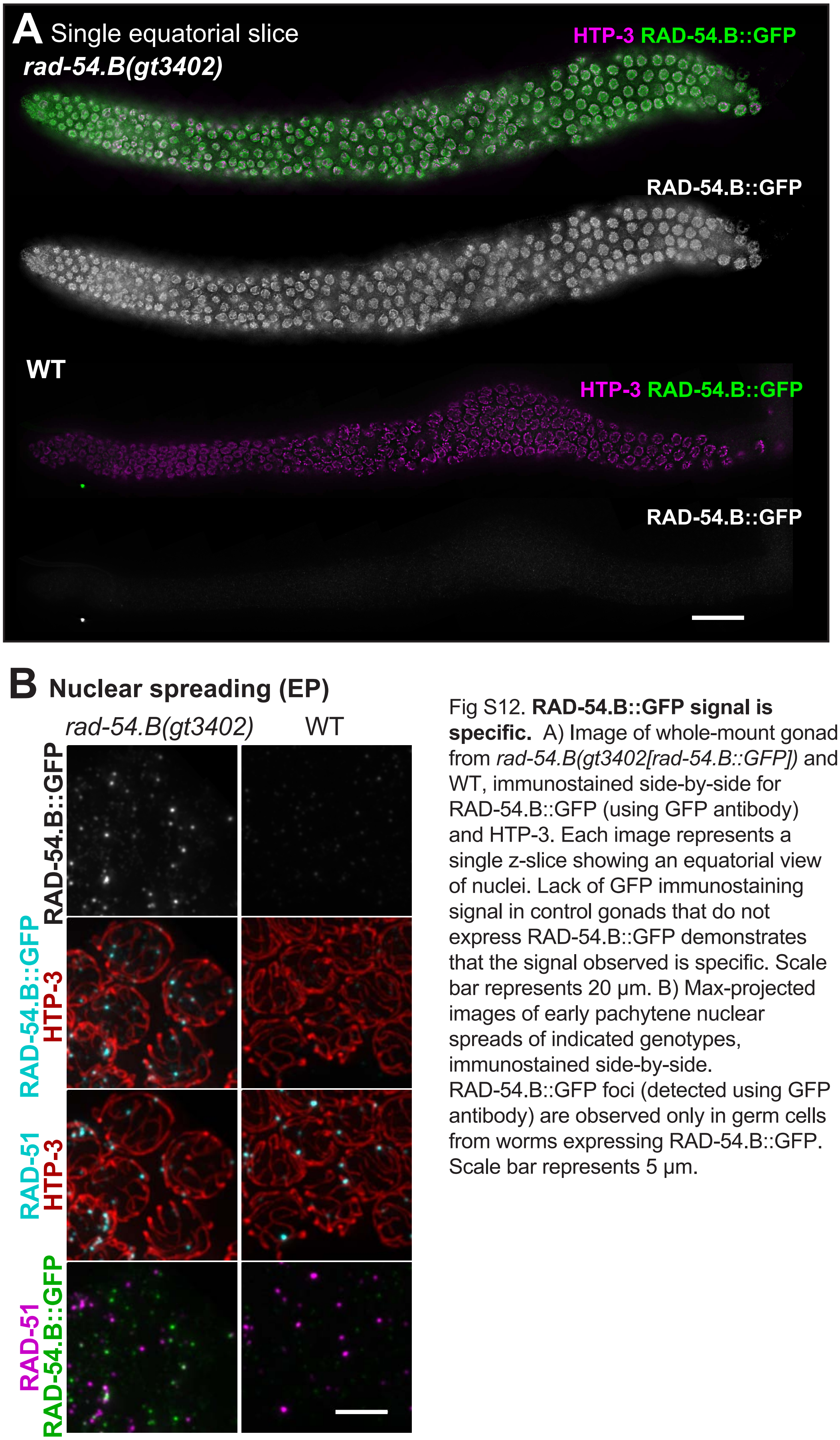

Fig S12. **RAD-54.B::GFP signal is specific.** A) Image of whole-mount gonad from *rad-54.B(gt3402[rad-54.B::GFP])* and WT, immunostained side-by-side for RAD-54.B::GFP (using GFP antibody) and HTP-3. Each image represents a single z-slice showing an equatorial view of nuclei. Lack of GFP immunostaining signal in control gonads that do not express RAD-54.B::GFP demonstrates that the signal observed is specific. Scale bar represents 20 µm. B) Max-projected images of early pachytene nuclear spreads of indicated genotypes, immunostained side-by-side. RAD-54.B::GFP foci (detected using GFP antibody) are observed only in germ cells from worms expressing RAD-54.B::GFP. Scale bar represents 5 µm.

Fig S13

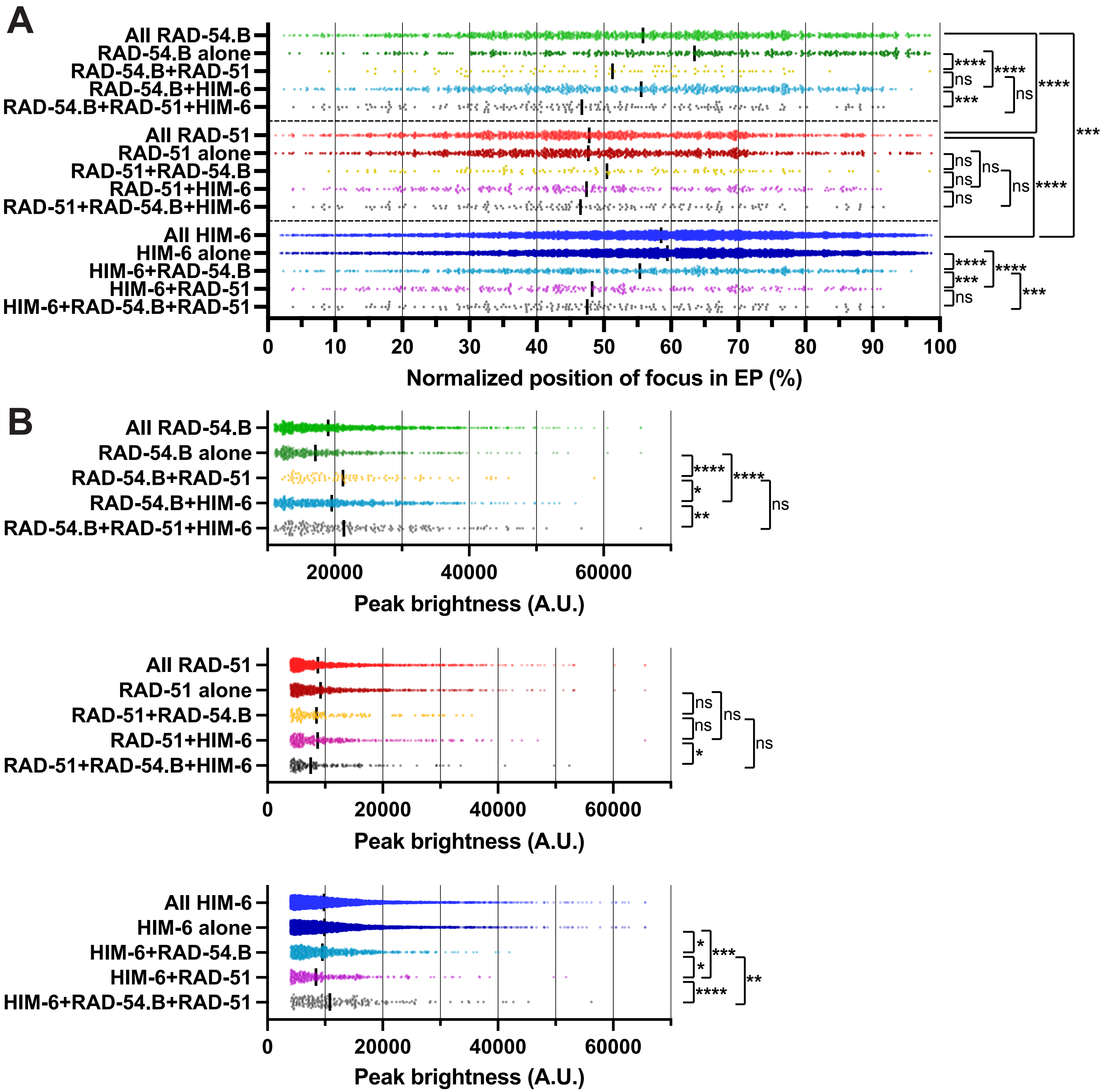
A
All RAD-54.B
RAD-54.B alone
RAD-54.B+RAD-51
RAD-54.B+HIM-6
RAD-54.B+RAD-51+HIM-6
All RAD-51
RAD-51 alone
RAD-51+RAD-54.B
RAD-51+HIM-6
RAD-51+RAD-54.B+HIM-6
All HIM-6
HIM-6 alone
HIM-6+RAD-54.B
HIM-6+RAD-51
HIM-6+RAD-54.B+RAD-51
0 10 20 30 40 50 60 70 80 90 100
Normalized position of focus in EP (%)
**** **** ns ns *** **** ***
ns ns ns ns ns ****
**** **** *** ns ***
B
All RAD-54.B
RAD-54.B alone
RAD-54.B+RAD-51
RAD-54.B+HIM-6
RAD-54.B+RAD-51+HIM-6
**** **** * ns **
20000 40000 60000
Peak brightness (A.U.)
All RAD-51
RAD-51 alone
RAD-51+RAD-54.B
RAD-51+HIM-6
RAD-51+RAD-54.B+HIM-6
ns ns ns ns *
0 20000 40000 60000
Peak brightness (A.U.)
All HIM-6
HIM-6 alone
HIM-6+RAD-54.B
HIM-6+RAD-51
HIM-6+RAD-54.B+RAD-51
* *** * ** ****
0 20000 40000 60000
Peak brightness (A.U.)

Fig S13. **Positions and peak brightness of RAD-54.B::GFP, RAD-51, and HIM-6 foci in early pachytene nuclear spreads.** A) Positions of early pachytene RAD-54.B::GFP, RAD-51, and HIM-6 foci with the indicated colocalization pattern, depicted as a percentage of the length of the total early pachytene zone. Early pachytene zone was defined as starting at the cell row in which all nuclei have a RAD-51 or HIM-6 focus (0%) and ending immediately before the cell row in which all nuclei have bright, CO-site associated HIM-6 foci (100%). Each point in the scatter plot represents a focus with the indicated colocalization pattern (alone, or with one or both other DSBR proteins) and the line indicates the median value. Statistical significance was assessed with a Mann Whitney test; ns, $p>0.05$; ***, $p<0.001$; ****, $p<0.0001$. B) Peak brightness values of early pachytene RAD-54.B::GFP, RAD-51, and HIM-6 foci with the indicated colocalization pattern. Each point in the scatter plot represents a focus with the indicated colocalization pattern and the line indicates the median value. Statistical significance was assessed with a Mann Whitney test; ns, $p>0.05$; *, $p<0.05$; **, $p<0.01$; ***, $p<0.001$; ****, $p<0.0001$.

Fig S14

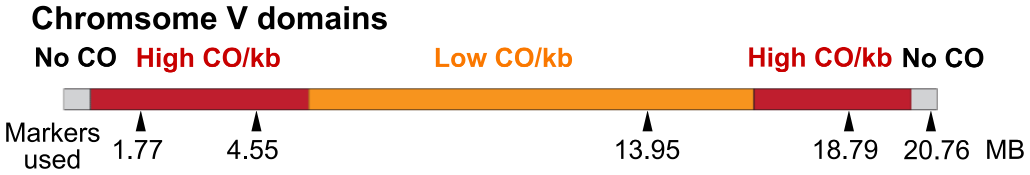

	Genotype	Interval 1	Interval 2	Interval 3	Interval 4	NCO	P-value
Expt 1	WT	29	18	34	12	99	0.034
	rad-54.B(null)	18	32	23	12	105	
Expt 2	WT	30	23	55	21	127	0.003
	rad-54.B(null)	14	40	34	15	153	

Fig S14. **The *rad-54.B(null)* mutant exhibits a modestly altered distribution of COs.** Top: schematic of chromosome V, with chromosome domains as defined by recombination assays (12) depicted by the colored boxes, and SNP markers used indicated with arrowheads below. Bottom: numbers of COs detected in each interval, and numbers of non-CO (NCO) products, from two independent experiments. P-value comparing CO distribution in WT vs *rad-54.B(gk340656)(null)* was calculated using a chi-square test for independence.
